# Supplementary material for: Understanding the psychological impact of the COVID-19 pandemic and containment measures: An empirical model of stress
Source: PLoS One. 2021 Jul 29;16(7):e0254883. doi: 10.1371/journal.pone.0254883 (PMC8320973; doi:10.1371/journal.pone.0254883)
Supplement: S1 Appendix — This appendix contains all materials and translations used in the survey, which were developed by the authors. (PDF) [file pone.0254883.s001.pdf]

Supporting Information Appendix S1

This document contains further information about the materials and methods used in the study. If there are any uncertainties, please do not hesitate to contact us (wissmath@psy.unibe.ch). All instructional texts and items are provided in German, French, and Italian. An English translation (not used in the survey) is provided as well. The Perceived Stress Scale (PSS) is not described, as it is a widely used scale whose documentation can be found in the corresponding literature. This document contains the following tables:

S1 Table A. Measurement of the amount of worries.

S1 Table B. Measurement of fear of COVID-19.

S1 Table C. Measurement of support.

S1 Table D. Measurement of agreement with containment measures.

S1 Table E. Measurement of positive aspects.

S1 Table F. Measurement of participants' individual situation and demographics.

Table A. The following table contains the instructional text and all items used to measure the amount of worries in German, French, and Italian. An English translation (not used in the survey) is provided.

**German:**					
Im Folgenden interessiert uns, ob Sie sich aufgrund der aktuellen Lage Sorgen zu bestimmten Themen machen. Wie sehr sorgen Sie sich um folgende Themen? Ich mache mir Sorgen um ...					
	gar nicht	wenig	mittel	stark	sehr stark
… meine körperliche Gesundheit					
… meine psychische Gesundheit					
… die Gesundheit meiner Freunde & Familie					
… meine Sicherheit					
… mein Sozialleben (Vereinsaktivität, Freizeitgestaltung, Hobbys)					
… mein Privatleben und meine persönlichen Bedürfnisse					
… meine finanzielle Situation					
… meinen Arbeitsplatz					
… die Wirtschaftslage					
… die Gesundheitsversorgung in der Schweiz					
… die Grundversorgung in der Schweiz (Lebensmittel, Güter des täglichen Bedarfs)					
**French:**					
Dans ce qui suit, nous aimerions savoir si vous vous faites du soucis à propos de certains thèmes à cause de la situation actuelle. À quel point vous faites-vous du soucis par rapport aux thèmes suivants ? Je me fais du souci à propos de...					
	pas du tout	un peu	moyen-nement	beaucoupt	énormé-ment
… ma santé physique					
… ma santé psychique					
… la santé de mes amis et de ma famille					
… ma sécurité					
… ma vie sociale (activités associatives, de loisirs et hobbys)					

… ma vie privée et mes besoins personnels					
… ma situation financière					
… mon lieu de travail					
… la situation économique					
… les soins de santé en Suisse					
… l'approvisionnement de base en Suisse (denrées alimentaires, articles de nécessité quotidienne)					
**Italian:**					
In seguito ci interessa se ha delle preoccupazioni che riguardano alcuni temi dovuti alla situazione attuale. Quanto si preoccupa dei seguenti aspetti?<br>Mi preoccupo per...					
	per niente	poco	medio	molto	moltissi-mo
… la mia salute fisica					
… la mia salute mentale					
… la salute dei miei amici e della mia famiglia					
… la mia sicurezza					
… la mia vita sociale (attività assiociative, tempo libero, hobby)					
… la mia vita privata e i miei bisogni individuali					
… la mia situazione finanziaria					
… il mio posto di lavoro					
… la situazione economica					
… l'assistenza sanitaria in Svizzera					
… i servizi di base in Svizzera (generi alimentari, beni di prima necessità)					
**English translation:**					
In the following, we are interested to know whether you are concerned about certain topics due to the current situation. How much do you worry about the following topics?<br>I worry about ...					
	not at all	little	medium	strongly	very strongly
… my physical health					

… my mental health					
… the health of my friends & family					
… my security					
… my social life (club activities, leisure activities, hobbies)					
… my private life and my personal needs					
… my financial situation					
… my job security					
… the economic situation					
… healthcare					
… the basic supply (food, goods of daily use)					

Note: Item "… my employment situation": This item was only presented if the participants of the survey had previously stated that they were currently employed.

Table B. The following table contains the items used to measure fear of COVID-19 in German, French, and Italian. An English translation (not used in the survey) is provided.

**German:**					
	gar nicht	wenig	mittel	viel	sehr viel
Wie viel Angst macht Ihnen das Coronavirus (COVID-19)?					
**French:**					
	pas du tout	un peu	moyen-nement	beaucoupt	énormé-ment
À quel point le coronavirus (Covid-19) vous effraie-t-il ?					
**Italian:**					
	per niente	poco	medio	molto	moltissi-mo
Quanta paura ha del coronavirus (COVID-19)?					
**English translation:**					
	not at all	little	medium	strongly	very strongly
How afraid are you of the coronavirus (COVID-19)?					

Table C. The following table contains the instructional text and the items used to measure support in German, French, and Italian. An English translation (not used in the survey) is provided.

**German:**						
Im Folgenden interessiert uns, durch wen Sie sich in der aktuellen Lage unterstützt fühlen.						
	gar nicht	wenig	teils-teils	stark	sehr stark	Kein Bedarf
Familie						
Freunde & Soziales Netzwerk						
Nachbarn / Mein Quartier						
Arbeitgeber						
Behörden						
Kindertagesstätten						
Schule / Ausbildungsstätte						
Kirche / Glaubensgemeinschaft						
Hausarzt						
Spitäler						
**French:**						
Dans ce qui suit, nous aimerions savoir par qui vous vous sentez soutenu pendant cette situation particulière.						
	pas du tout	peu	partiel-lement	beaucoup	énormé-ment	pas de besion
Famille						
Amis & réseau social						
Voisins / mon quartier						
Employeur						
Autorités						
La crèche						
École / centre de formation						

Église / communauté religieuse						
Médecin traitant						
Hôpitaux						
**Italian:**						
Di seguito ci interessa da chi si sente sostenuto nella situazione attuale.						
	per niente	poco	in parte	molto	moltissi-mo	non ne ho bisogno
Famiglia						
Amici & rete sociale						
Vicini / quartiere						
Datore di lavoro						
Le autorità						
Asili nido						
Scuola/ Centro di formazione						
Chiesa / Comunità religiosa						
Medico di famiglia						
Ospedali						
**English translation:**						
In the following, we would like to know who you perceive as a support in the current situation.						
	not at all	Little	partially	strongly	very strongly	no need
Family						
Friends & social network						
Neighbors / my neighborhood						
Employer						
Authorities						
Day-care centers						
School						

Church / religious community						
Primary care physician						
Hospitals						

Notes: 1) Item "Employer": This item was only presented if the participants of the survey previously stated that they were currently employed. 2) To compute the score, "no need", "not at all", and "little" were coded as 0, "partially" was coded as 1, "strongly" as 2, and "very strongly" as 3.

Table D. The following table contains the item used to measure agreement with containment measures in German, French, and Italian. An English translation (not used in the survey) is provided.

**German:**
Wie schätzen Sie die aktuellen Massnahmen der Behörden ein?<br>Die Massnahmen...<br><br>… gehen viel zu wenig weit<br>… gehen zu wenig weit<br>… sind genau richtig<br>… gehen zu weit<br>… gehen viel zu weit
**French:**
Comment trouvez-vous les mesures prises par les autorités ?<br>Les mesures...<br><br>… ne vont pas du tout assez loin<br>… ne vont pas assez loin<br>… sont tout à fait appropriées<br>… vont trop loin<br>… vont beaucoup trop loin
**Italian:**
Come valuta le misure adottate dalle autorità?<br>Le misure sono...<br><br>… molto insufficienti<br>… insufficienti<br>… proprio giuste<br>… eccessive<br>… molto eccessive
**English translation:**
How do you assess the current measures taken by the authorities?<br>The measures are...<br><br>… not at all strict enough<br>… not strict enough<br>… just right<br>… too strict<br>… much too strict

Table E. The following table contains the item asking whether there are any positive aspects in German, French, and Italian. An English translation (not used in the survey) is provided.

**German:**
Gibt es in der aktuellen Lage auch positive Aspekte für Sie? (Ja / Nein)
**French:**
Selon vous, y a-t-il aussi des aspects positifs à la situation actuelle ? (Oui / Non)
**Italian:**
Secondo lei ci sono anche aspetti positivi nella situazione attuale? (Si / No)
**English translation:**
Are there any positive aspects for you in the current situation? (Yes / No)

Table F. The following table contains further information about how participants' individual situation and demographics were measured. If specific answer items were used, they are provided in German, French, and Italian. An English translation (not used in the survey) is either provided or can be retrieved from Table 1 of the main article. For material that is not further described in the following table (e.g. measurement of "having kids"), we believe that the materials are sufficiently described in the main article.

**Education:**		
**German:**<br>Welches ist Ihr höchster Bildungsabschluss?<br><br>- Ich habe noch keinen Schulabschluss<br>- obligatorische Primar- und Oberstufe<br>- Lehre, Berufsschule, Handelsschule<br>- Kantonsschule, Gymnasium<br>- Höhere Fach- oder Berufsschule<br>- Universität, Hochschule<br>- Sonstiges: (Texteingabefeld)	**French:**<br>Quel est votre niveau d'étude le plus élevé ?<br><br>- Je n'ai pas encore terminé l'école obligatoire<br>- École obligatoire<br>- Apprentissage, école professionnelle ou de commerce<br>- Gymnase, lycée, collège<br>- École professionnelle ou technique supérieure<br>- Université, haute école<br>- Autre : (champ de saisie de texte)	**Italian:**<br>Qual'è il suo livello d'istruzione più alto?<br><br>- Non ho ancora un diploma scolastico<br>- Scuola dell'obbligo<br>- Apprendistato, scuola di formazione, scuola commerciale<br>- Liceo, ginnasio<br>- Scuola professionale superiore<br>- Università, scuole universitarie<br>- Altro: (campo di inserimento testo)
**Type of household:**		
**German:**<br>In welcher Wohnform wohnen Sie?<br><br>- Einpersonenhaushalt<br>- Nichtfamilienhaushalt mit mehreren Personen<br>- Paar ohne Kind(er) im Haushalt<br>- Paar mit Kind(er) im Haushalt<br>- Elternteil mit Kindern im Haushalt<br>- Mehrfamilienhaushalt<br>- Alters- oder Pflegeheim	**French:**<br>Dans quelle sorte de ménage vivez-vous ?<br><br>- Ménage d'une personne<br>- Ménage non familial de plusieurs personnes<br>- Couple sans enfant<br>- Couple avec enfant(s)<br>- Père ou mère seul-e avec enfant(s)<br>- Ménage plurifamilial<br>- Maison de retraite ou maison de repos	**Italian:**<br>In che tipo di economia domestica vive?<br><br>- Economia domestica di una persona<br>- Economia domestica pluricomponente non familiare<br>- Coppia senza figli<br>- Coppia con figli<br>- Genitori soli con figlio/i<br>- Economia domestica plurifamiliare<br>- Casa per anziani o casa di cura

Part of a risk group:

By the time of the survey, being part of a risk group was defined by the swiss authorities by the following criteria:

- Being 65 years old or older
- Being adult and having one of the following pre-existing conditions:
    - High blood pressure
    - Diabetes
    - Cardiovascular Disease
    - Respiratory Disease
    - Cancer

As the authorities potentially could have changed these criteria during the survey, they were not listed in the survey and participants were given the opportunity to respond with "don't know".

Media consumption:

**German:**	**French:**	**Italian:**
Wie oft informieren Sie sich in den Medien zum aktuellen Stand der Lage?<br><br>- Nie<br>- 1-2 Mal pro Woche<br>- 3-4 Mal pro Woche<br>- 1 Mal pro Tag<br>- Mehrmals pro Tag	À quelle fréquence vous informez-vous dans les médias au sujet de l'état de la situation actuelle ?<br><br>- Jamais<br>- 1-2 fois par semaine<br>- 3-4 fois par semaine<br>- 1 fois par jour<br>- Plusieurs fois par jour	Con quale frequenza consulta i media per informarsi sullo stato attuale della situazione?<br><br>- Mai<br>- 1-2 volte a settimana<br>- 3-4 volte a settimana<br>- 1 volta al giorno<br>- Più volte al giorno

English translation of instructional text: "How often do you read up on the current situation in the media?"
Answer-Items: See Table 1 of the main article.

Estimated length of crisis:

**German:**	**French:**	**Italian:**
Wie lange denken Sie, wird die Epidemie in der Schweiz noch dauern?<br><br>- Zwei Wochen<br>- Einen Monat<br>- Zwei Monate<br>- 3-5 Monate<br>- Ein halbes Jahr<br>- Ein Jahr<br>- Die Epidemie wird noch länger als ein Jahr dauern	À votre avis, combien de temps l'épidémie va-t-elle encore durer en Suisse ?<br><br>- Deux semaines<br>- Un mois<br>- Deux mois<br>- 3-5 mois<br>- Une demi année<br>- Un an<br>- L'épidémie durera plus d'un an	Quanto pensa che durerà l'epidemia in Svizzera?<br><br>- due settimane<br>- un mese<br>- due mesi<br>- 3-5 mesi<br>- mezz'anno<br>- un anno<br>- L'epidemia durerà più di un anno

English translation of instructional text: "How long do you think the epidemic will last in Switzerland?"
Answer-Items: See Table 1 of the main article.
