# Supplementary material for: Understanding the psychological impact of the COVID-19 pandemic and containment measures: An empirical model of stress
Source: PLoS One. 2021 Jul 29;16(7):e0254883. doi: 10.1371/journal.pone.0254883 (PMC8320973; doi:10.1371/journal.pone.0254883)
Supplement: Attachment — Submitted filename: PONE-D-20-16793_R1_reviewer11.pdf [file pone.0254883.s003.pdf]

PLOS ONE

Understanding the psychological impact of the COVID-19 pandemic and containment measures: an empirical model of stress.

--Manuscript Draft--

**Manuscript Number:**	PONE-D-20-16793R1
**Article Type:**	Research Article
**Full Title:**	Understanding the psychological impact of the COVID-19 pandemic and containment measures: an empirical model of stress.
**Short Title:**	Psychological impact of the COVID-19 pandemic and containment measures
**Corresponding Author:**	Bartholomäus Wissmath
**Keywords:**	COVID-19; containment measures; psychological impact; empirical model; Stress
**Abstract:**	Epidemics such as COVID-19 and corresponding containment measures are assumed to cause psychological stress. In a survey during the lockdown in Switzerland (n=1565), we found substantially increased levels of stress in the population. In particular, individuals who did not agree with the containment measures, as well as those who saw nothing positive in the crisis, experienced high levels of stress. In contrast, individuals who are part of a risk group or who are working in healthcare or in essential shops experienced similar stress levels as the general public. The psychological mechanisms that determine stress, caused by the COVID-19 pandemic and containment measures, are not yet clear. Thus, we conducted a path analysis to gain a deeper understanding of the psychological mechanisms that lead to stress. Experiencing fear of the disease is a key driver for being worried. Our model further shows that worries about the individual, social, and economic consequences of the crisis, strongly boost stress. The infection rate in the canton (i.e. state) of residence also contributes to stress. Positive thinking and perceived social, organizational, and governmental support mitigate worries and stress. Our findings indicate that containment measures increase worries and stress, especially for those who feel that these measures are either not sufficient or go too far. Thus, highlighting positive aspects of the crisis and convincing people of the effectiveness and necessity of mitigation measures can, not only promote compliance, but also reduce stress. Our model suggests that people who feel protected by the authorities have fewer worries, which can, in turn limit the negative impact of the crisis on mental health.
**Order of Authors:**	Bartholomäus Wissmath
	Fred W Mast
	Fabian Kraus
	David Weibel
**Response to Reviewers:**	Reply to Reviewer 1’s comments<br>Reviewer 1 writes that our manuscript is “well written, with clear explanations and use of data”, with only one suggestion for improvement pertaining to the abstract. We have addressed this issue in the following reply.<br>Reviewer 1: “The only thing I would suggest rewriting is the abstract. The conclusion is alive and hard hitting, but somehow the abstract isn't. Maybe the first sentence could be stronger; ‘Research suggests’ is a very woolly start.”<br><br>Reply: We agree with the reviewer that the abstract could be improved and have therefore revised it. The beginning of the abstract has been modified and the conclusions restructured to give weight on the impact of our findings. The revised abstract now reads as follows:<br>“Epidemics such as COVID-19 and corresponding containment measures are assumed to cause psychological stress. In a survey during the lockdown in Switzerland (n=1565), we found substantially increased levels of stress in the population. In particular, individuals who did not agree with the containment measures, as well as those who saw nothing positive in the crisis, experienced high levels of stress. In contrast, individuals who are part of a risk group or who are working in healthcare or in

Powered by Editorial Manager® and ProduXion Manager® from Aries Systems Corporation

essential shops experienced similar stress levels as the general public. The psychological mechanisms that determine stress, caused by the COVID-19 pandemic and containment measures, are not yet clear. Thus, we conducted a path analysis to gain a deeper understanding of the psychological mechanisms that lead to stress. Experiencing fear of the disease is a key driver for being worried. Our model further shows that worries about the individual, social, and economic consequences of the crisis, strongly boost stress. The infection rate in the canton (i.e. state) of residence also contributes to stress. Positive thinking and perceived social, organizational, and governmental support mitigate worries and stress. Our findings indicate that containment measures increase worries and stress, especially for those who feel that these measures are either not sufficient or go too far. Thus, highlighting positive aspects of the crisis and convincing people of the effectiveness and necessity of mitigation measures can, not only promote compliance, but also reduce stress. Our model suggests that people who feel protected by the authorities have fewer worries, which can, in turn limit the negative impact of the crisis on mental health."

Reply to Reviewer 2's comments

Reviewer 2 states that our study is "technically-sound, well-executed […] with clear explanation of research methods and sound statistical analysis." Furthermore, he or she notes that there are no ethical concerns, that the data are available, and that the conclusions are appropriate.

Reply: We appreciate Reviewer 2's positive evaluation of our manuscript and we are grateful for his or her feedback.

Reply to Reviewer 3's comments

Reviewer 3 concludes that our study is interesting. However, he or she raises some questions and provides suggestions for improvement. In the following section, we present Reviewer 3's recommendations to improve the paper and address each point in detail.

Reviewer 3: When throughout the Introduction section, the authors correctly reported that COVID-19 evokes stress and that reduced social interactions are risk factors for mental disorders such as major depression, they could even mention the association between stress, depression and negative outcomes. In particular, the unique sensory processing patterns of individuals with major affective disorders and their relationship with psychiatric symptomatology and clinicial outcomes have been reported. Hyposensitivity or hypersensitivity may be "trait" markers of individuals with major affective disorders and interventions should refer to the individual unique sensory profiles and their behavioral and functional impact in the context of real life. Thus, given the above information, I suggest to cite, within the main text, the following papers about this topic (PMID: 28855878, 31103905, 31031655). In addition, subjects with a history of childhood maltreatment may be even at increased risk of negative outcomes such as suicidal behavior. Specifically, the relation between childhood maltreatment, depression, and suicidal behaviors has been demonstrated. Importantly, the exposure to abuse and neglect as a child may enhance the risk to develop both symptoms of depression and higher suicidal risk. Thus, in order to briefly discuss this topic (although I understand that the link between depression, childhood maltreatment, and suicidal behavior is not the main topic of the present manuscript), my suggestion is to cite, within the main text, the following additional manuscripts (PMID: 25169890; 32824995; 30551339).

Reply: We thank the reviewer for these suggestions. We write in our paper that certain individuals are particularly vulnerable to consequences of the pandemic and the containment measures. We agree with the reviewer that existing research shows that alexithymia, childhood maltreatment, as well as neuroticism, can be such risk factors. In the revised version, we mention this:
"Some individuals may be more vulnerable to psychosocial consequences; risk factors include certain personality traits (e.g. neuroticism, alexithymia), mental or physical pre-existing conditions, as well as previous traumatic experiences (e.g. childhood maltreatment), or the individual's current situation, such as working in healthcare,

Powered by Editorial Manager® and ProduXion Manager® from Aries Systems Corporation

	social isolation, or poverty [4-10]" (see manuscript without track changes, p. 3, line 39ff)<br>We agree with the reviewer that a comprehensive exploration of the impact of childhood maltreatment and alexithymia es somewhat beyond the scope of our study, but we find the inputs useful and have incorporated them later in the discussion. Our study shows that individuals who do not see anything positive about the pandemic show strongly increased stress levels. The question arises as to how this can be explained. Since childhood maltreatment is associated with hopelessness and lack of perspective, it is possible that the experience of maltreatment in the past could play a role. We have incorporated this into the conclusion, the corresponding passage reads as follows:<br>"Although seeing something positive about the crisis may be associated with the individual situation, personality traits, and the respondent's coping behavior [17], our findings indicate that recognizing positive aspects could alleviate stress. Some individuals, however, do not see anything positive about the crisis. This raises the question as to why their assessment of the situation is so negative. For example, childhood maltreatment is associated with hopelessness [20] and psychological distress [5]. A recent study in connection with COVID-19 indicates that pre-pandemic maltreatment makes people particularly vulnerable to negative psychosocial consequences of the pandemic [21]. In addition, other factors and traits – such as pessimistic thinking [e.g. 22] or generalized anxiety [e.g. 23] – could account for the lack of perspective." (see manuscript without track changes, p. 7, line 110ff)<br>Furthermore, it is now mentioned in the manuscript (limitation section) that it would be promising to examine the role of alexithymia in further studies (see also reply to answer to point 4).<br><br>Additional references in the revised version (in the same order as they appear in the article):<br>Liu S, Lithopoulos A, Zhang CQ, Garcia-Barrera MA, Rhodes RE. Personality and perceived stress during COVID-19 pandemic: Testing the mediating role of perceived threat and efficacy. Pers Individ Dif. 2021;168:110351. Epub 2020 Aug 21.<br>De Berardis D, Fornaro M, Orsolini L, Valchera A, Carano A, Vellante F et al. Alexithymia and suicide risk in psychiatric disorders: a mini-review. Front Psychiatry. 2017;8:148.<br>Pompili M, Innamorati M, Lamis DA, Erbuto D, Venturini P, Ricci F, Serafini G, Amore M, Girardi P. The associations among childhood maltreatment, "male depression" and suicide risk in psychiatric patients. Psychiatry Res. 2014;220(1-2):571-8.<br>Santiago, C. D., Wadsworth, M. E., & Stump, J. (2011). Socioeconomic status, neighborhood disadvantage, and poverty-related stress: Prospective effects on psychological syndromes among diverse low-income families. Journal of Economic Psychology, 32(2), 218-230.<br>Wanklyn SG, Day DM, Hart TA, Girard TA. Cumulative childhood maltreatment and depression among incarcerated youth: impulsivity and hopelessness as potential intervening variables. Child Maltreat. 2012;17(4):306-17.<br>Guo J, Fu M, Liu D, Zhang B, Wang X, van IJzendoorn MH. Is the psychological impact of exposure to COVID-19 stronger in adolescents with pre-pandemic maltreatment experiences? A survey of rural Chinese adolescents. Child Abuse Negl. 2020;20:104667.<br>Norem JK. Defensive pessimism, optimism, and pessimism. In: Chang EC, editor, Optimism & pessimism: Implications for theory, research, and practice. Washington DC: American Psychological Association; 2001. p. 77–100.<br>Miranda R, Mennin DS. Depression, generalized anxiety disorder, and certainty in pessimistic predictions about the future. Cognit Ther Res. 2007;31(1),71-82.<br>Betsch, C. How behavioural science data helps mitigate the COVID-19 crisis. Nat Hum Behav. 2020;4:438.<br>Andrews JL, Foulkes L, Blakemore SJ. Peer Influence in Adolescence: Public-Health Implications for COVID-19. Trends Cogn Sci. 2020;24(8):585-587.<br>Tang W, Hu T, Yang L, Xu J. The role of alexithymia in the mental health problems of home-quarantined university students during the COVID-19 pandemic in China. Pers Individ Dif. 2020;165:110131.<br><br>Reviewer 3: The most relevant aims/objectives and study hypotheses of the present study need to be better described within the main text.

Powered by Editorial Manager® and ProduXion Manager® from Aries Systems Corporation

Reply: We agree with the reviewer that the aim of the study could be better explained. We now describe this in more detail in the revised version. Here is the relevant text passage:
“However, little is known about the psychological mechanisms that determine individual stress in this crisis. It is still unclear which individuals are particularly affected by stress, which factors have an increasing or decreasing influence on stress, and how these factors interact. In particular, the interplay between fear of the virus, infection rate, risk factors, individual and collective resources, perception of the containment strategy, and psychological stress has not yet been fully understood. The aim of the present study is to address these questions by using path analysis.” (see manuscript without track changes, p. 3, line 52ff)

Reviewer 3: While the authors reported that they investigated a total of 1565 individuals who completed the online survey, they did not specify how many individuals were excluded to participate. In addition, the most relevant psychometric instruments reported in the present manuscript could be described more succinctly.

Reply: The number of participants refers to those who completed the survey and did not request the deletion of their data. 215 individuals had not completed or had cancelled the survey. The data of three participants were removed since they had requested the deletion of their data after completion of the survey. This is now described in the manuscript. We reassessed the description of the psychometric instruments and made it more succinct wherever possible. However, we would like to mention that the editor (see below) has asked us to provide sufficient information regarding the survey and questionnaire items.

Reviewer 3: The major shortcomings/limitations of this paper need to be better discussed as the description of the main caveats has been not included within the main text.

Reply: We thank the reviewer for bringing this point to our attention. We have now added a passage at the end of the Conclusion section, which addresses limitations and possible future studies. We also mention that it would be useful to examine the role of alexithymia and neuroticism in more detail. The additional passage reads as follows:
“There are some limitations to the study. First, the cross-sectional design of the study limited the ability to make inferences about the directions of causality. Second, the sample is not representative in terms of gender; as in most studies using the PSS, women are somewhat overrepresented. Third, we could not assess all individual characteristics that could have an influence on stress levels. For example, neuroticism or alexithymia could be relevant traits that make individuals particularly vulnerable to crises such as a pandemic [26]. It would be interesting to investigate the role of these characteristics in future studies.” (see manuscript without track changes, p. 9, line 145ff)

Reviewer 3: What is the take-home message of the present manuscript? While the authors reported that providing support is an important way to mitigate worries, enable coping, and reduce stress, they could provide some additional conclusive remarks about this topic. Specifically, how worries and stress might be really mitigated? How protective coping strategies can be implemented? Here, more details/information are needed.

Reply: The take-home message is that the pandemic significantly increases stress levels and that this increase depends on a number of factors, with support, communicating positive aspects, and advocacy of the authorities' measures as being particularly important. We believe that we have already clearly stated this in the manuscript. However, we agree with the reviewer that we could conclude the discussion with additional suggestions on what could be done to mitigate stress. We have included a corresponding passage in the revised manuscript:
“Taken together, to mitigate stress, authorities should explain containment measures well, highlight positive aspects of the crisis, address worries, and facilitate support. Since agreement with the containment measures may decrease stress, it is crucial that the measures are well-explained and their importance emphasized, and that measures are backed up by scientific evidence. Since the protection of others is an important motivating factor to restrict one's own everyday life [24], the authorities could thus

	emphasize that compliance with containment measures helps to protect those in need of protection. Furthermore, our analyses show that support through communities, relatives, and employers seems to be key in preventing stress; thus, these support networks should be encouraged to provide help. We identified young individuals as a group experiencing high stress levels. A recent study suggests that social media are particularly well-suited to reach out to adolescents [25]. Since we show that worries are a key driver for stress, our study also provides arguments for economic support, such as stimulus checks or short-time work. In addition, our study has identified groups that are particularly affected by the crisis. This information could help to distribute resources and target efforts." (see manuscript without track changes, p. 8, line 131ff)<br><br>Reply to additional comments from the Editor<br><br>The editorial office further mentions two additional requirements that need to be addressed.<br><br>Editor: Please ensure that your manuscript meets PLOS ONE's style requirements, including those for file naming.<br><br>Reply: As requested, it is ensured that all formal requirements are met.<br><br>Editor: Please include additional information regarding the survey or questionnaire used in the study and ensure that you have provided sufficient details that others could replicate the analyses. For instance, if you developed a questionnaire as part of this study and it is not under a copyright more restrictive than CC-BY, please include a copy, in both the original language and English, as Supporting Information.<br><br>Reply: As requested, we have added some additional information in order to provide sufficient details (see appendix S1).
**Additional Information:**	
**Question**	**Response**
**Financial Disclosure**<br><br>Enter a financial disclosure statement that describes the sources of funding for the work included in this submission. Review the submission guidelines for detailed requirements. View published research articles from *PLOS ONE* for specific examples.<br><br>This statement is required for submission and **will appear in the published article** if the submission is accepted. Please make sure it is accurate.	The authors received no specific funding for this work.

**Unfunded studies**<br>Enter: *The author(s) received no specific funding for this work.*<br><br>**Funded studies**<br>Enter a statement with the following details:<br>• Initials of the authors who received each award<br>• Grant numbers awarded to each author<br>• The full name of each funder<br>• URL of each funder website<br>• Did the sponsors or funders play any role in the study design, data collection and analysis, decision to publish, or preparation of the manuscript?<br>• **NO** - Include this sentence at the end of your statement: *The funders had no role in study design, data collection and analysis, decision to publish, or preparation of the manuscript.*<br>• **YES** - Specify the role(s) played.<br><br>* typeset	
**Competing Interests**<br><br>Use the instructions below to enter a competing interest statement for this submission. On behalf of all authors, disclose any competing interests that could be perceived to bias this work—acknowledging all financial support and any other relevant financial or non-financial competing interests.<br><br>This statement **will appear in the published article** if the submission is accepted. Please make sure it is accurate. View published research articles from *PLOS ONE* for specific examples.	The authors have declared that no competing interests exist.

Powered by Editorial Manager® and ProduXion Manager® from Aries Systems Corporation

**NO authors have competing interests**<br><br>Enter: *The authors have declared that no competing interests exist.*<br><br>**Authors with competing interests**<br><br>Enter competing interest details beginning with this statement:<br><br>*I have read the journal's policy and the authors of this manuscript have the following competing interests: [insert competing interests here]*<br><br>* typeset	
**Ethics Statement**<br><br>Enter an ethics statement for this submission. This statement is required if the study involved:<br><br>• Human participants<br>• Human specimens or tissue<br>• Vertebrate animals or cephalopods<br>• Vertebrate embryos or tissues<br>• Field research<br><br>Write "N/A" if the submission does not require an ethics statement.<br><br>General guidance is provided below. Consult the submission guidelines for detailed instructions. **Make sure that all information entered here is included in the Methods section of the manuscript.**	The study was reviewed and approved by the Ethics Committee of the Faculty of Human Sciences, University of Bern.

Powered by Editorial Manager® and ProduXion Manager® from Aries Systems Corporation

**Format for specific study types**<br><br>**Human Subject Research (involving human participants and/or tissue)**<br>• Give the name of the institutional review board or ethics committee that approved the study<br>• Include the approval number and/or a statement indicating approval of this research<br>• Indicate the form of consent obtained (written/oral) or the reason that consent was not obtained (e.g. the data were analyzed anonymously)<br><br>**Animal Research (involving vertebrate animals, embryos or tissues)**<br>• Provide the name of the Institutional Animal Care and Use Committee (IACUC) or other relevant ethics board that reviewed the study protocol, and indicate whether they approved this research or granted a formal waiver of ethical approval<br>• Include an approval number if one was obtained<br>• If the study involved *non-human primates*, add *additional details* about animal welfare and steps taken to ameliorate suffering<br>• If anesthesia, euthanasia, or any kind of animal sacrifice is part of the study, include briefly which substances and/or methods were applied<br><br>**Field Research**<br><br>Include the following details if this study involves the collection of plant, animal, or other materials from a natural setting:<br>• Field permit number<br>• Name of the institution or relevant body that granted permission	
**Data Availability**<br><br>Authors are required to make all data underlying the findings described fully available, without restriction, and from the time of publication. PLOS allows rare exceptions to address legal and ethical concerns. See the PLOS Data Policy and FAQ for detailed information.	Yes - all data are fully available without restriction

A Data Availability Statement describing where the data can be found is required at submission. Your answers to this question constitute the Data Availability Statement and **will be published in the article**, if accepted.<br><br>**Important:** Stating 'data available on request from the author' is not sufficient. If your data are only available upon request, select 'No' for the first question and explain your exceptional situation in the text box.<br><br>Do the authors confirm that all data underlying the findings described in their manuscript are fully available without restriction?	
**Describe where the data may be found in full sentences. If you are copying our sample text, replace any instances of XXX with the appropriate details.**<br><br>• If the data are **held or will be held in a public repository**, include URLs, accession numbers or DOIs. If this information will only be available after acceptance, indicate this by ticking the box below. For example: *All XXX files are available from the XXX database (accession number(s) XXX, XXX.*).<br>• If the data are all contained **within the manuscript and/or Supporting Information files**, enter the following: *All relevant data are within the manuscript and its Supporting Information files.*<br>• If neither of these applies but you are able to provide **details of access elsewhere**, with or without limitations, please do so. For example:<br><br>*Data cannot be shared publicly because of [XXX]. Data are available from the XXX Institutional Data Access / Ethics Committee (contact via XXX) for researchers who meet the criteria for access to confidential data.*<br><br>*The data underlying the results presented in the study are available from (include the name of the third party*	Understanding the psychological impact of the COVID-19 pandemic and containment measures: an empirical model of stress [Dataset]. Available from: https://osf.io/grvwa/. Deposited May 13, 2020.

and contact information or URL).<br>• This text is appropriate if the data are owned by a third party and authors do not have permission to share the data.<br><br>* typeset	
Additional data availability information:	

Powered by Editorial Manager® and ProduXion Manager® from Aries Systems Corporation

Manuscript Click here to access/download;Manuscript;Manuscript.docx

Understanding the psychological impact of the COVID-19 pandemic and containment measures: an empirical model of stress

Bartholomäus Wissmath [1,2,3*], Fred W. Mast [1,3], Fabian Kraus[1,2], David Weibel [1,2,3]

[1] Institute of Psychology, University of Bern, Fabrikstrasse 8, 3012 Bern, Switzerland

[2] w hoch 2 GmbH, Kramgasse 5, 3011 Bern, Switzerland

[3] Swiss Distance Learning University, Überlandstrasse 12, 3900 Brig, Switzerland

* Corresponding author.

E-mail: wissmath@psy.unibe.ch

Abstract

Epidemics such as COVID-19 and corresponding containment measures are assumed to cause psychological stress. In a survey during the lockdown in Switzerland (n=1565), we found substantially increased levels of stress in the population. In particular, individuals who did not agree with the containment measures, as well as those who saw nothing positive in the crisis, experienced high levels of stress. In contrast, individuals who are part of a risk group or who are working in healthcare or in essential shops experienced similar stress levels as the general public. The psychological mechanisms that determine stress, caused by the COVID-19 pandemic and containment measures, are not yet clear. Thus, we conducted a path analysis to gain a deeper understanding of the psychological mechanisms that lead to stress. Experiencing fear of the disease is a key driver for being worried. Our model further shows that worries about the individual, social, and economic consequences of the crisis, strongly boost stress. The infection rate in the canton (i.e. state) of residence also contributes to stress. Positive thinking and perceived social, organizational, and governmental support mitigate worries and stress. Our findings indicate that containment measures increase worries and stress, especially for those who feel that these measures are either not sufficient or go too far. Thus, highlighting positive aspects of the crisis and convincing people of the effectiveness and necessity of mitigation measures can, not only promote compliance, but also reduce stress. Our model suggests that people who feel protected by the authorities have fewer worries, which can, in turn limit the negative impact of the crisis on mental health.

Introduction

COVID-19 evokes stress in patients, healthcare professionals, and relatives [1]. The impact may not be limited to those directly affected; health worries and uncertainty are assumed to generate fear, anxiety, and severe stress in the general population [2]. Hence, mental health practitioners anticipate a sharp rise in the need for mental health services [3]. Some individuals may be more vulnerable to psychosocial consequences; risk factors include certain personality traits (e.g. neuroticism, alexithymia) mental or physical pre-existing conditions, as well as previous traumatic experiences (e.g. childhood maltreatment), or the individual's current situation such as working in healthcare, social isolation, or poverty [4-10].

Switzerland was among the countries first and most affected by COVID-19 in Europe. To curb the spread of the virus, the Swiss government enacted a set of containment measures: encouraging hygiene, giving shelter-in-place orders, as well as closing borders, child-care facilities, schools, restaurants, bars, leisure facilities, and non-essential shops. Events and gatherings of more than five individuals were banned.

These measures directly affect the general population and may come with side effects. Reduced social interactions are risk factors for mental disorders such as major depression [11], and the containment measures may trigger an economic downturn, thus adding further stress [12]. Early studies on COVID-19 observed increased stress levels [13]. However, little is known about the psychological mechanisms that determine individual stress in this crisis. It unclear which individuals are particularly affected by stress, which factors have an increasing or decreasing influence on stress, and how these factors interact. In particular, the interplay between fear of the virus, infection rate, risk factors, individual and collective resources, perception of the containment strategy, and psychological stress has not yet been fully understood. The aim of the present study

is to address these questions by using path analysis. We assume that fear of COVID-19 and the local infection rate trigger stress [14,15]. Individual, economic, and societal worries are also expected to increase stress levels. Agreement with containment measures, perceived support, [14,16], and being optimistic about the crisis [17], are likely to decrease stress. During the lockdown, we surveyed 1565 individuals in Switzerland online.

Results

First, we compared the observed stress levels in our sample (M=15.58; SD=6.65) to a representative community sample under normal conditions (M=12.57; SD=6.24) [18]. A one-sample Gauss test showed higher stress levels during the lockdown period, z=19.08, p <.001. The effect (d=.48) is medium to strong.

In a second step, we computed multiple comparisons (ANOVA) of different subgroups (see Table 1). Women expressed higher stress levels than men, as has been found in non-epidemic situations [19]. Young individuals reported the highest stress levels, whereas the levels were lowest for individuals older than 65 years (small to medium effect). The majority of respondents (66%) agreed with the measures of the Swiss authorities. These individuals scored lower on stress than individuals who felt that the measures were either not sufficient or too extreme. Individuals who strongly disagreed experienced even more stress (medium effect). Individuals who reported that the lockdown situation also has positive aspects (89.4%) had substantially lower stress levels compared to those who did not see any positive aspects at all (strong effect[illegible]

Table 1: Group comparisons of experienced stress

Variable	Sample statistics (N = 1565)			ANOVA statistics		
	n	Mean PSS Score	SD	F	p	Effect size
Sex^{+}				12.6	<.001*	$\eta^2 = 0.016$
(A) Female	1043	16.1	6.24			
(B) Male	516	14.4	6.84			
(C) Other / not specified	6	17.5	10.4			
Age^{+}				8.84	<.001*	$\eta^2 = 0.028$
(A) <25	126	17.4	6.87			
(B) 25-34	371	16.1	6.10			
(C) 35-44	380	16.2	6.29			
(D) 45-54	295	14.9	6.78			
(E) 55-64	261	15.1	6.67			
(F) > 64	132	12.9	5.95			
Education				2.66	0.014	$\eta^2 = 0.01$
(A) no finished education	5	13.2	4.02			
(B) mandatory school time (9 years)	40	15.8	6.77			
(C) apprenticeship, vocational school, commercial school	311	15.3	6.97			
(D) High School	99	17.6	6.9			
(E) Higher technical or vocational school	298	14.8	6.28			
(F) University	778	15.6	6.28			
(G) Other	34	16.7	6.94			
Type of household				2.19	0.042	$\eta^2 = 0.008$
(A) Single person household	327	15.5	7.14			
(B) Non-family household with multiple persons	127	15.8	6.05			
(C) Couple without kids in the household	440	14.8	6.29			
(D) Couple with kids in the household	469	15.8	6.14			
(E) Single parent with kids in the household	143	16.7	6.86			
(F) Multiple-family household	58	15.4	6.93			
(G) Retirement or nursing home	1	25.0	N/A (n = 1)			
Having Kids				1.58	0.209	d = 0.077
Yes	472	15.9	6.33			
No	1093	15.4	6.58			
Employment at the beginning of crisis				0.59	0.442	d = -0.046
Yes	1252	15.5	6.31			
No	313	15.8	7.24			
Working in healthcare[1)]				0.42	0.516	d = 0.048
Yes	235	15.7	5.82			
No	1017	15.4	6.42			
Working in an essential shop[1)]				0.83	0.361	d = 0.111
Yes	91	16.1	5.57			
No	1161	15.4	6.36			
Part of a risk group				4.77	0.009	$\eta^2 = 0.006$
(A) Yes	265	15.4	7.27			
(B) No	1180	15.4	6.30			
(C) Don't know	120	17.3	6.52			
Agreement with government's containment strategy^{+}				48.8	<.001*	$\eta^2 = 0.036$
(A) Agreement	1036	14.8	6.10			
(B) No agreement	425	16.7	6.46			
(C) No agreement at all	104	18.8	8.55			
Following shelter-in-place orders stringently				3.19	0.074	d = 0.108
Yes	1041	15.8	6.53			
No	524	15.1	6.44			
News consumption				3.23	0.012	$\eta^2 = 0.008$
(A) Never	15	17.1	8.20			
(B) 1-2 times per week	126	14.6	6.53			
(C) 3-4 times per week	157	15.5	5.81			
(D) Once per day	634	15.1	6.21			
(E) Multiple times per day	633	16.2	6.86			
Estimated duration of the crisis				0.90	0.496	$\eta^2 = 0.003$
(A) 2 weeks	30	14.8	7.40			
(B) 1 month	163	14.8	6.32			
(C) 2 months	555	15.6	6.47			
(D) 3-5 months	484	15.8	6.20			
(E) 6 months	161	15.1	6.45			
(F) 1 year	74	15.8	7.23			
(G) longer than a year	98	16.2	7.60			
Seeing positive aspects of the crisis				87.6	<.001*	d = 0.767
Yes	1415	15.1	6.15			
No	150	20.2	7.85			

p = significance of ANOVA main effects; **+** = significant ($p < .001$) post hoc Bonferroni tests: Sex: (A) > (B), Age: (A) > (F), Agreement with containment strategy: (A) < (B) < (C); ***** = significant after Bonferroni correction ($p < 0.00357$); [1)] unemployed participants were excluded from analysis.

No other assessed variable influenced stress (cf. Table 1). Interestingly, individuals identifying as members of a risk group, as well as individuals working in healthcare or in essential shops with customer contact, did not experience more stress than the rest of the population. Also, the expected duration of the pandemic and amount of news consumption had no influence on stress

In a third step, we conducted a path analysis with stress as the target variable. Fig. 1 depicts the resulting model, showing a good fit.

Fig 1. A path model showing the influence of various determinants on stress. Model fit statistics: χ^2 (10) = 42.85, p < .01, GFI = .99, CFI = .97, NFI = .96, TLI = .94, RMSEA = .05. All displayed paths are highly significant (p < .01.). Error variances appear in small circles.

In total, the measured variables explain 30% of variance in the endogenous variable, stress. Worries contribute the most to stress, and worries mediate between fear of COVID-19 and stress. Seeing positive aspects of the crisis, as well as agreement with the government's containment strategy, are correlated, and both mitigate worries and boost perceived support. In addition, positive thinking directly minimizes stress. Perceived support from family, friends, organizations, and authorities mitigates worries and reduces stress.

Discussion

Our results show that the COVID-19 pandemic and the mitigation strategies increase the population's stress level. We present a model that builds upon existing theories of stress and explains 30% of the variance in stress.

In our model, fear of the virus is the most important booster of worries and the amount of worries is the most important driver of stress. Agreeing with the authorities' containment strategy and seeing positive aspects of the crisis are important factors mitigating stress. Correspondingly, feeling that the containment measures are not sufficient or too extreme is associated with more stress. Support provided by family, friends, organizations, and authorities are also important protective factors for stress.

In our study, risk factors for high stress levels are a high amount of worries and perceived lack of support and, to a lesser extent, high infection rates. Eleven percent of the respondents do not see anything positive about the crisis. This group experiences substantially more stress than individuals who see at least one positive aspect. Although seeing something positive about the crisis may be associated with the individual situation, personality traits, and respondent's coping behavior [17], our findings indicate that recognizing positive aspects alleviates stress. Some individuals, however, do not see anything positive about the crisis. This raises the question as to why their assessment of the situation is so negative. For example, childhood maltreatment is associated with hopelessness [20] and psychological distress [5]. A recent study in connection with COVID-19 indicates that pre-pandemic maltreatment makes people particularly vulnerable to negative psychosocial consequences of the pandemic [21]. In addition, other factors and traits – such as pessimistic thinking [e.g. 22] or generalized anxiety [e.g. 23] – could account for the lack of perspective.Surprisingly, members of the risk groups, old respondents and individuals working in healthcare or essential shops with customer contact, did not score higher on stress. One reason could be that extensive protection measures had been mandated. In addition, healthcare providers in Switzerland have so far not been overwhelmed by cases of COVID-19.

Our model could be useful in understanding and addressing the psychological impact of possible new waves of COVID-19 cases and other epidemics. Mitigation measures boost worries and stress, particularly for those individuals who feel that these measures are not sufficient or go too far. Highlighting positive aspects about the crisis and convincing people of the effectiveness and the necessity of containment measures may not only boost compliance, but also decrease stress, since individuals feel protected by the authorities and experience less worries. This, in turn, will hopefully limit the impact on mental health as a consequence of the crisis. Providing support is another important way to mitigate worries, enable coping, and reduce stress.

Taken together, to mitigate stress, authorities should explain containment measures well, highlight positive aspects of the crisis, address worries, and facilitate support. Since agreement with the containment measures may decrease stress, it is crucial that the measures are well-explained and their importance emphasized, and that measures are backed up by scientific evidence. Since the protection of others is an important motivating factor to restrict one's own everyday life [24], the authorities could thus emphasize that compliance with containment measures helps to protect those in need of protection. Furthermore, our analyses show that support through communities, relatives, and employers seems to be key in preventing stress; thus, these support networks should be encouraged to provide help. We identified young individuals as a group experiencing high stress levels. A recent study suggests that social media are particularly well-suited to reach out to adolescents [25]. Since we show that worries are a key driver for stress, our study also provides arguments for economic support, such as stimulus checks or short-time work. In addition, our study has identified groups that are particularly affected by the crisis. This information could help to distribute resources and target efforts.

There are some limitations to the study. First, the cross-sectional design of the study limited the ability to make inferences about the directions of causality. Second, the sample is not representative in terms of gender; as in most studies using the PSS, women are somewhat overrepresented. Third, we could not assess all individual characteristics that could have an influence on stress levels. For example, neuroticism or alexithymia could be relevant traits that make individuals particularly vulnerable to crises such as a pandemic [26]. It would be interesting to investigate the role of these characteristics in future studies.

Materials and methods

Participants

From March 27 to April 26, 2020, a total of 1565 individuals completed the online survey. 215 individuals had not completed or had cancelled the survey. The data of three participants were removed since they had requested the deletion of their data after completion of the survey.Participants were recruited via academic institutions and social media. All participants were Swiss residents. Information about the sample is provided in Table 1. Individuals from Geman-speaking (n=1206), French-speaking (n=247) and Italian-speaking (n=112) regions, were surveyed. The study was reviewed and approved by the Ethics Committee of the Faculty of Human Sciences, University of Bern.

Measurements

Stress

We used the German [27], French [28], and Italian [29] version of the Perceived Stress Scale (PPS-10) (10-items) [18, 19]. The widely-used PSS is based on the stress-related components

of unpredictable, uncontrollable, and overloading life events (e.g. "In the last month, how often have you been angry because of things that were outside your control?"), (0=never; 4=very often). A PSS score was calculated by summing up all items. Reliability was high (Chronbach's alpha = .86).

Amount of worries

We assessed worries about the following aspects (1=not at all; 5=a lot): physical health, mental health, health of family and friends, security, social life, private life and personal needs, financial situation, job security, economic situation, healthcare, and basic supply. A principal component analysis on these items suggest a one-factorial solution. We computed the mean value across all items.

Fear of COVID-19

Fear was measured with a single item: "How afraid are you of the coronavirus (COVID-19)?" (1=not at all; 5=very strongly).

Support

Participants assessed perceived support from: family, friends and social network, neighbors, employer, authorities, day-care centers, schools, church or religious community, primary care physician, hospitals (0=little or no support; 3=strong support). To compute the score, we summed up the ratings.

Infection rate

The cases per 100,000 inhabitants in the participants' resident canton (state) were used [30].

Agreement with containment measures

We asked the participants whether they thought that the measures were (a) not at all strict enough (b) not strict enough (c) just right (d) too strict (e) much too strict. For the analyses, the categories (a) and (e) were combined as “no agreement at all,” and the categories (b) and (d) were combined as “no agreement.” These combined categories were contrasted with (c) “agreement.”

Seeing positive aspects

We asked the respondents whether there were any positive aspects for them in the current situation (1=No; 2=Yes).

Participants’ individual situation and demographics

We assessed sex, age, education, type of household, having kids, employment, working in healthcare or essential shops with customer interaction, part of a risk group, media consumption, and estimated length of the crisis.

Data Availab[illegible]y

Replication data are available on the Open Science Framework [31], https://osf.io/grvwa/

References

1. Brooks SK, Webster RK, Smith LE, Woodland L, Wessely S, Greenberg N, et al. The psychological impact of quarantine and how to reduce it: rapid review of the evidence. The Lancet. 2020;395(10227):912-20.
2. Pfefferbaum B, North CS. Mental Health and the Covid-19 Pandemic. N Engl J Med. 2020;383:510-512.
3. Fiorillo A, Gorwood P. The consequences of the COVID-19 pandemic on mental health and implications for clinical practice. Eur. Psychiatry. 2020;63(1):e32,1-2.

4. Liu S, Lithopoulos A, Zhang CQ, Garcia-Barrera MA, Rhodes RE. Personality and perceived stress during COVID-19 pandemic: Testing the mediating role of perceived threat and efficacy. Pers Individ Dif. 2021;168:110351. Epub 2020 Aug 21.
5. De Berardis D, Fornaro M, Orsolini L, Valchera A, Carano A, Vellante F et al. Alexithymia and suicide risk in psychiatric disorders: a mini-review. Front Psychiatry. 2017;8:148.
6. Pompili M, Innamorati M, Lamis DA, Erbuto D, Venturini P, Ricci F, Serafini G, Amore M, Girardi P. The associations among childhood maltreatment, "male depression" and suicide risk in psychiatric patients. Psychiatry Res. 2014;220(1-2):571-8.
7. Limcaoco RSG, Mateos EM, Fernandez JM, Roncero C. Anxiety, worry and perceived stress in the world due to the COVID-19 pandemic, March 2020. Preliminary results. medRxiv [Preprint]. 2020 [cited 2020 Jun 2]. Available from: https://www.medrxiv.org/content/10.1101/2020.04.03.20043992v1
8. Pellecchia U, Crestani R, Decroo T, Van den Bergh R, Al-Kourdi Y. Social Consequences of Ebola Containment Measures in Liberia. PLoS One. 2015;10(12):e0143036.
9. Varshney M, Parel JT, Raizada N, Sarin SK. Initial psychological impact of COVID-19 and its correlates in Indian Community: An online (FEEL-COVID) survey. PLoS One. 2020;15(5):e0233874.
10. Santiago CD, Wadsworth ME, Stump J. Socioeconomic status, neighborhood disadvantage, and poverty-related stress: Prospective effects on psychological syndromes among diverse low-income families. J Econ Psychol. 2011;32(2), 218-230.

11. Bruce ML, Hoff RA. Social and physical health risk factors for first-onset major depressive disorder in a community sample. Soc. Psychiatry. Psychiatr. Epidemiol. 1994;29(4):165-71.
12. Mehta K, Kramer H, Durazo-Arvizu R, Cao G, Tong L, Rao M. Depression in the US population during the time periods surrounding the great recession. J. Clin. Psychiatry. 2015;76(4):e499-504.
13. Wang C, Pan R, Wan X, Tan Y, Xu L, Ho CS, et al. Immediate Psychological Responses and Associated Factors during the Initial Stage of the 2019 Coronavirus Disease (COVID-19) Epidemic among the General Population in China. Int J Environ Res Public Health. 2020;17(5):1729.
14. Kelloway EK, Mullen J, Francis L. The Stress (of an) Epidemic. Stress and Health. 2012;28(2):91–7.
15. Barbisch D, Koenig KL, Shih F-Y. Is There a Case for Quarantine? Perspectives from SARS to Ebola. Disaster Med. Public Health Prep. 2015;9(5):547-53.
16. Wong TY, Koh GC, Cheong SK, Fong YT, Sundram M, Koh K. Concerns, Perceived Impact and Preparedness in an Avian Influenza Pandemic – a Comparative Study between Healthcare Workers in Primary and Tertiary Care. Ann Acad Med Singapore. 2008;37(2):7.
17. Naseem Z, Khalid R. Positive Thinking in Coping with Stress and Health outcomes: Literature Review. Journal of Research & Reflections in Education (JRRE). 2010;4(1):42-61.

18. Klein EM, Brähler E, Dreier M, Reinecke L, Müller KW, Schmutzer G, et al. The German version of the Perceived Stress Scale – psychometric characteristics in a representative German community sample. BMC Psychiatry. 2016;16(1):159.

19. Cohen S., Williamson G. Perceived stress in a probability sample of the U.S. In: S. Spacapam S, Oskamp S, editors. The social psychology of health: Claremont Symposium on Applied Social Psychology. Newbury Park, CA: Sage Publications; 1988 p. 31-67.

20. Wanklyn SG, Day DM, Hart TA, Girard TA. Cumulative childhood maltreatment and depression among incarcerated youth: impulsivity and hopelessness as potential intervening variables. Child Maltreat. 2012;17(4):306-17.

21. Guo J, Fu M, Liu D, Zhang B, Wang X, van IJzendoorn MH. Is the psychological impact of exposure to COVID-19 stronger in adolescents with pre-pandemic maltreatment experiences? A survey of rural Chinese adolescents. Child Abuse Negl. 2020;20:104667.

22. Norem JK. Defensive pessimism, optimism, and pessimism. In: Chang EC, editor, Optimism & pessimism: Implications for theory, research, and practice. Washington DC: American Psychological Association; 2001. p. 77–100.

23. Miranda R, Mennin DS. Depression, generalized anxiety disorder, and certainty in pessimistic predictions about the future. Cognit Ther Res. 2007;31(1),71-82.

24. Betsch, C. How behavioural science data helps mitigate the COVID-19 crisis. Nat Hum Behav. 2020;4:438.

25. Andrews JL, Foulkes L, Blakemore SJ. Peer Influence in Adolescence: Public-Health Implications for COVID-19. Trends Cogn Sci. 2020;24(8):585-587.

26. Tang W, Hu T, Yang L, Xu J. The role of alexithymia in the mental health problems of home-quarantined university students during the COVID-19 pandemic in China. Pers Individ Dif. 2020;165:110131.
27. Schneider EE, Schönfelder S, Domke-Wolf M, Wessa M. Measuring stress in clinical and nonclinical subjects using a German adaptation of the Perceived Stress Scale. Int J Clin Health Psychol. 2020;20(2):173-181.
28. Ben Loubir D, Serhier Z, Otmani N, Housbane S, Mouddene NA, Agoub M, Bennani Othmani M. Le stress perçu: validation de la traduction d'une échelle de mesure de stress en dialecte marocain [Perceived stress: validation of the translation of a stress measuring scale in Moroccan dialect]. Pan Afr Med J. 2015; 12;21:280.
29. Fossati A. Traduzione Italiana della scala per lo stress percepito [Italian translation of the Perceived Stress Scale]. 2010 [cited 2020 October 15]. Available from: http://www.pensierocritico.eu/files/Italian_PSS_10_with_info.pdf
30. Probst D. COVID-19 Information for Switzerland. 2020 [cited 2020 June 2]. Available from: https://www.corona-data.ch/
31. Wissmath B, Mast FW, Kraus F, Weibel D. Understanding the psychological impact of the COVID-19 pandemic and containment measures: an empirical model of stress [Dataset]. Available from: https://osf.io/grvwa/. Deposited May 13, 2020.

Supporting Information

S1 Appendix. Detailed description of materials and methods. This appendix contains all materials used in the survey, which were developed by the authors.

Figure

Click here to access/download;Figure;Fig1.tif

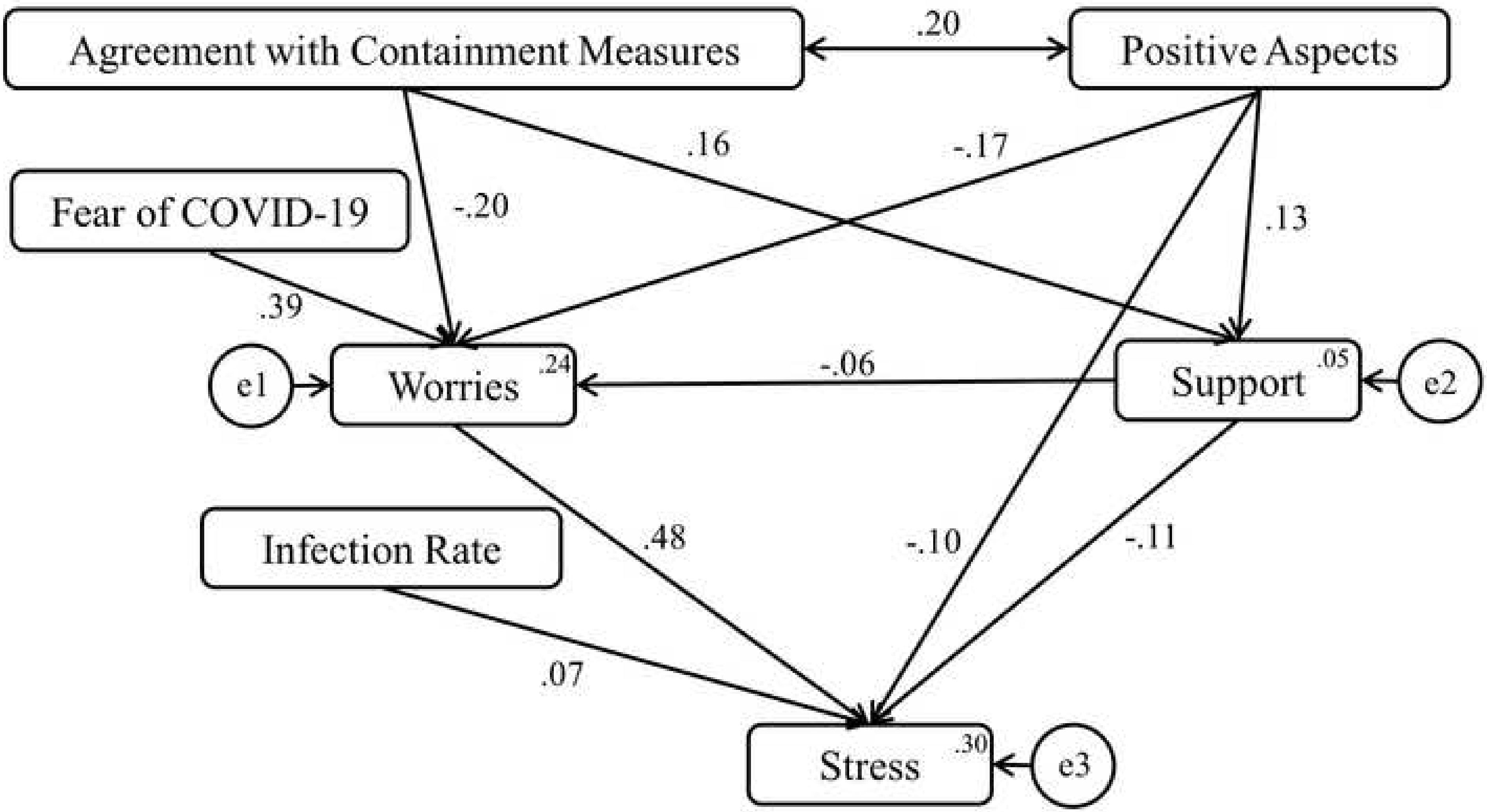

Click here to access/download
Supporting Information
Appendix S1.pdf

Understanding the psychological impact of the COVID-19 pandemic and containment measures: an empirical model of stress

Bartholomäus Wissmath [1,2,3*], Fred W. Mast [1,3], Fabian Kraus[1,2], David Weibel [1,2,3]

[1] Institute of Psychology, University of Bern, Fabrikstrasse 8, 3012 Bern, Switzerland

[2] w hoch 2 GmbH, Kramgasse 5, 3011 Bern, Switzerland

[3] Swiss Distance Learning University, Überlandstrasse 12, 3900 Brig, Switzerland

* Corresponding author.

E-mail: wissmath@psy.unibe.ch

Abstract

~~Research suggests that e~~Epidemics such as COVID-19 and corresponding containment measures ~~have negative consequences to the individual and~~are assumed to cause psychological stress. ~~The psychological mechanisms that determine stress, caused by the COVID-19 pandemic and containment measures, are not yet clear.~~ In a survey during the lockdown in Switzerland (n=1565), we found substantially increased levels of stress in the population. In particular, individuals who did not agree with the containment measures, as well as those who saw nothing positive in the crisis, experienced ~~even higher~~high levels of stress. In contrast, individuals who are part of a risk group or who are working in healthcare or in essential shops experienced similar stress levels as the general public. The psychological mechanisms that determine stress, caused by the COVID-19 pandemic and containment measures, are not yet clear. Thus, w~~W~~e conducted a path analysis to gain a deeper understanding of the psychological mechanisms ~~during lockdown~~that lead to stress. Experiencing fear of the disease is a key driver for being worried. Our model further shows that worries about the individual, social, and economic consequences of the crisis, strongly boost stress. The infection rate in the canton (i.e. state) of residence also contributes to stress. Positive thinking and perceived social, organizational, and governmental support mitigate worries and stress. Our findings indicate that containment measures increase worries and stress, especially for those who feel that these measures are either not sufficient or go too far. Thus, highlighting positive aspects of the crisis and convincing people of the effectiveness and necessity of mitigation measures can, not only promote compliance, but also reduce stress. Our model suggests that people who feel protected by the authorities have fewer worries, which can, in turn limit the negative impact of the crisis on mental health.~~To prevent stress, authorities should explain~~

~~containment measures well, highlight positive aspects of the crisis, address worries, and facilitate support.~~

Introduction

COVID-19 evokes stress in patients, healthcare professionals, and relatives [1]. The impact may not be limited to those directly affected; health worries and uncertainty are assumed to generate fear, anxiety, and severe stress in the general population [2]. Hence, mental health practitioners anticipate a sharp rise in the need for mental health services [3]. Some individuals may be more vulnerable to psychosocial consequences; risk factors include certain personality traits (e.g. neuroticism, alexithymia) mental or physical pre-existing conditions, as well as previous traumatic experiences (e.g. childhood maltreatment), or the individual's current situation such as working in healthcare, ~~or~~ social isolation, or poverty [4-10].

Switzerland was among the countries first and most affected by COVID-19 in Europe. To curb the spread of the virus, the Swiss government enacted a set of containment measures: encouraging hygiene, giving shelter-in-place orders, as well as closing borders, child-care facilities, schools, restaurants, bars, leisure facilities, and non-essential shops. Events and gatherings of more than five individuals were banned.

These measures directly affect the general population and may come with side effects. Reduced social interactions are risk factors for mental disorders such as major depression [11~~7~~], and the containment measures may trigger an economic downturn, thus adding further stress [12~~8~~]. Early studies on COVID-19 observed increased stress levels [13~~9~~]. However, little is known about the psychological mechanisms that determine individual stress in this crisis. It unclear which individuals are particularly affected by stress, which factors have an increasing or decreasing

influence on stress, and how these factors interact. In particular, the interplay between fear of the virus, infection rate, risk factors, individual and collective resources, perception of the containment strategy, and psychological stress has not yet been fully understood. The aim of the present study is to address these questions by using path analysis. We assume that fear of COVID-19 and the local infection rate trigger stress [14~~0~~,15~~1~~]. Individual, economic, and societal worries are also expected to increase stress levels. Agreement with containment measures, perceived support, [14~~0~~,16~~2~~], and being optimistic about the crisis [17~~3~~], are likely to decrease stress. During the lockdown, we surveyed 1565 individuals in Switzerland online.

Results

First, we compared the observed stress levels in our sample (*M*=15.58; *SD*=6.65) to a representative community sample under normal conditions (*M*=12.57; *SD*=6.24) [18~~4~~]. A one-sample Gauss test showed higher stress levels during the lockdown period, *z*=19.08, p <.001. The effect (d=.48) is medium to strong.

In a second step, we computed multiple comparisons (ANOVA) of different subgroups (see Table 1). Women expressed higher stress levels than men, as has been found in non-epidemic situations [19~~5~~]. Young individuals reported the highest stress levels, whereas the levels were lowest for individuals older than 65 years (small to medium effect). The majority of respondents (66%) agreed with the measures of the Swiss authorities. These individuals scored lower on stress than individuals who felt that the measures were either not sufficient or too extreme. Individuals who strongly disagreed experienced even more stress (medium effect). Individuals who reported that the lockdown situation also has positive aspects (89.4%) had substantially lower stress levels compared to those who did not see any positive aspects at all (strong effect).

Table 1: Group comparisons of experienced stress

Variable	Sample statistics (N = 1565)			ANOVA statistics		
	n	Mean PSS Score	SD	F	p	Effect size
Sex^{+}				12.6	<.001*	η^2 = 0.016
(A) Female	1043	16.1	6.24			
(B) Male	516	14.4	6.84			
(C) Other / not specified	6	17.5	10.4			
Age^{+}				8.84	<.001*	η^2 = 0.028
(A) <25	126	17.4	6.87			
(B) 25-34	371	16.1	6.10			
(C) 35-44	380	16.2	6.29			
(D) 45-54	295	14.9	6.78			
(E) 55-64	261	15.1	6.67			
(F) > 64	132	12.9	5.95			
Education				2.66	0.014	η^2 = 0.01
(A) no finished education	5	13.2	4.02			
(B) mandatory school time (9 years)	40	15.8	6.77			
(C) apprenticeship, vocational school, commercial school	311	15.3	6.97			
(D) High School	99	17.6	6.9			
(E) Higher technical or vocational school	298	14.8	6.28			
(F) University	778	15.6	6.28			
(G) Other	34	16.7	6.94			
Type of household				2.19	0.042	η^2 = 0.008
(A) Single person household	327	15.5	7.14			
(B) Non-family household with multiple persons	127	15.8	6.05			
(C) Couple without kids in the household	440	14.8	6.29			
(D) Couple with kids in the household	469	15.8	6.14			
(E) Single parent with kids in the household	143	16.7	6.86			
(F) Multiple-family household	58	15.4	6.93			
(G) Retirement or nursing home	1	25.0	N/A (n = 1)			
Having Kids				1.58	0.209	d = 0.077
Yes	472	15.9	6.33			
No	1093	15.4	6.58			
Employment at the beginning of crisis				0.59	0.442	d = -0.046
Yes	1252	15.5	6.31			
No	313	15.8	7.24			
Working in healthcare[1)]				0.42	0.516	d = 0.048
Yes	235	15.7	5.82			
No	1017	15.4	6.42			
Working in an essential shop[1)]				0.83	0.361	d = 0.111
Yes	91	16.1	5.57			
No	1161	15.4	6.36			
Part of a risk group				4.77	0.009	η^2 = 0.006
(A) Yes	265	15.4	7.27			
(B) No	1180	15.4	6.30			
(C) Don't know	120	17.3	6.52			
Agreement with government's containment strategy^{+}				48.8	<.001*	η^2 = 0.036
(A) Agreement	1036	14.8	6.10			
(B) No agreement	425	16.7	6.46			
(C) No agreement at all	104	18.8	8.55			
Following shelter-in-place orders stringently				3.19	0.074	d = 0.108
Yes	1041	15.8	6.53			
No	524	15.1	6.44			
News consumption				3.23	0.012	η^2 = 0.008
(A) Never	15	17.1	8.20			
(B) 1-2 times per week	126	14.6	6.53			
(C) 3-4 times per week	157	15.5	5.81			
(D) Once per day	634	15.1	6.21			
(E) Multiple times per day	633	16.2	6.86			
Estimated duration of the crisis				0.90	0.496	η^2 = 0.003
(A) 2 weeks	30	14.8	7.40			
(B) 1 month	163	14.8	6.32			
(C) 2 months	555	15.6	6.47			
(D) 3-5 months	484	15.8	6.20			
(E) 6 months	161	15.1	6.45			
(F) 1 year	74	15.8	7.23			
(G) longer than a year	98	16.2	7.60			
Seeing positive aspects of the crisis				87.6	<.001*	d = 0.767
Yes	1415	15.1	6.15			
No	150	20.2	7.85			

p = significance of ANOVA main effects; **+** = significant ($p < .001$) post hoc Bonferroni tests: Sex: (A) > (B), Age: (A) > (F), Agreement with containment strategy: (A) < (B) < (C); ***** = significant after Bonferroni correction ($p < 0.00357$); [1)] unemployed participants were excluded from analysis.

No other assessed variable influenced stress (cf. Table 1). Interestingly, individuals identifying as members of a risk group, as well as individuals working in healthcare or in essential shops with customer contact, did not experience more stress than the rest of the population. Also, the expected duration of the pandemic and amount of news consumption had no influence on stress.

In a third step, we conducted a path analysis with stress as the target variable. Fig. 1 depicts the resulting model, showing a good fit.

Fig 1. A path model showing the influence of various determinants on stress. Model fit statistics: χ^2 (10) = 42.85, p < .01, GFI = .99, CFI = .97, NFI = .96, TLI = .94, RMSEA = .05. All displayed paths are highly significant (p < .01.). Error variances appear in small circles.

In total, the measured variables explain 30% of variance in the endogenous variable, stress. Worries contribute the most to stress, and worries mediate between fear of COVID-19 and stress. Seeing positive aspects of the crisis, as well as agreement with the government’s containment strategy, are correlated, and both mitigate worries and boost perceived support. In addition, positive thinking directly minimizes stress. Perceived support from family, friends, organizations, and authorities mitigates worries and reduces stress.

Discussion

Our results show that the COVID-19 pandemic and the mitigation strategies increase the population’s stress level. We present a model that builds upon existing theories of stress and explains 30% of the variance in stress.

In our model, fear of the virus is the most important booster of worries and the amount of worries is the most important driver of stress. Agreeing with the authorities' containment strategy and seeing positive aspects of the crisis are important factors mitigating stress. Correspondingly, feeling that the containment measures are not sufficient or too extreme is associated with more stress. Support provided by family, friends, organizations, and authorities are also important protective factors for stress.

In our study, risk factors for high stress levels are a high amount of worries and perceived lack of support and, to a lesser extent, high infection rates. Eleven percent of the respondents do not see anything positive about the crisis. This group experiences substantially more stress than individuals who see at least one positive aspect. Although seeing something positive about the crisis may be associated with the individual situation, personality traits, and respondent's coping behavior [17~~13~~], our findings indicate that recognizing positive aspects alleviates stress. ~~Our model builds upon existing theories of stress and explains 30% of the variance in stress.~~ Some individuals, however, do not see anything positive about the crisis. This raises the question as to why their assessment of the situation is so negative. For example, childhood maltreatment is associated with hopelessness [20] and psychological distress [5]. A recent study in connection with COVID-19 indicates that pre-pandemic maltreatment makes people particularly vulnerable to negative psychosocial consequences of the pandemic [21]. In addition, other factors and traits – such as pessimistic thinking [e.g. 22] or generalized anxiety [e.g. 23] – could account for the lack of perspective.

Surprisingly, members of the risk groups, old respondents and individuals working in healthcare or essential shops with customer contact, did not score higher on stress. One reason

could be that extensive protection measures had been mandated. In addition, healthcare providers in Switzerland have so far not been overwhelmed by cases of COVID-19.

Our model could be useful in understanding and addressing the psychological impact of possible new waves of COVID-19 cases and other epidemics. Mitigation measures boost worries and stress, particularly for those individuals who feel that these measures are not sufficient or go too far. Highlighting positive aspects about the crisis and convincing people of the effectiveness and the necessity of containment measures may not only boost compliance, but also decrease stress, since individuals feel protected by the authorities and experience less worries. This, in turn, will hopefully limit the impact on mental health as a consequence of the crisis. Providing support is another important way to mitigate worries, enable coping, and reduce stress. Taken together, to mitigate stress, authorities should explain containment measures well, highlight positive aspects of the crisis, address worries, and facilitate support. Since agreement with the containment measures may decrease stress, it is crucial that the measures are well-explained and their importance emphasized, and that measures are backed up by scientific evidence. Since the protection of others is an important motivating factor to restrict one's own everyday life [24], the authorities could thus emphasize that compliance with containment measures helps to protect those in need of protection. Furthermore, our analyses show that support through communities, relatives, and employers seems to be key in preventing stress; thus, these support networks should be encouraged to provide help. We identified young individuals as a group experiencing high stress levels. A recent study suggests that social media are particularly well-suited to reach out to adolescents [25]. Since we show that worries are a key driver for stress, our study also provides arguments for economic support, such as stimulus checks or short-time work. In

addition, our study has identified groups that are particularly affected by the crisis. This information could help to distribute resources and target efforts.

There are some limitations to the study. First, the cross-sectional design of the study limited the ability to make inferences about the directions of causality. Second, the sample is not representative in terms of gender; as in most studies using the PSS, women are somewhat overrepresented. Third, we could not assess all individual characteristics that could have an influence on stress levels. For example, neuroticism or alexithymia could be relevant traits that make individuals particularly vulnerable to crises such as a pandemic [26]. It would be interesting to investigate the role of these characteristics in future studies.

Materials and methods

Participants

From March 27 to April 26, 2020, a total of 1565 individuals completed the online survey. 215 individuals had not completed or had cancelled the survey. The data of three participants were removed since they had requested the deletion of their data after completion of the survey. Participants were recruited via academic institutions and social media. All participants were Swiss residents. Information about the sample is provided in Table 1. Individuals from Geman-speaking (n=1206), French-speaking (n=247) and Italian-speaking (n=112) regions, were surveyed. The study was reviewed and approved by the Ethics Committee of the Faculty of Human Sciences, University of Bern.

Measurements

Stress

We used the German [27], French [28], and Italian [29] version of the Perceived Stress Scale (PPS-10) (10-items) [18~~4~~, 19~~5~~]. The widely-used PSS is based on the stress-related components of unpredictable, uncontrollable, and overloading life events (e.g. “In the last month, how often have you been angry because of things that were outside your control?”), (0=never; 4=very often). A PSS score was calculated by summing up all items~~, with a high score indicating high stress levels~~. Reliability was high (Chronbach’s alpha = .86).

Amount of worries

~~Participants evaluated to what extent they were worried~~We assessed worries about the following aspects (1=not at all; 5=a lot): physical health, mental health, health of family and friends, security, social life, private life and personal needs, financial situation, job security,

economic situation, healthcare, and basic supply. A principal component analysis on these items suggest a one-factorial solution. ~~For our analyses, w~~We ~~thus~~ computed the mean value across all items.

Fear of COVID-19

Fear was measured with ~~one~~ a single item: “How afraid are you of the coronavirus (COVID-19)?” (1=not at all; 5=very strongly).

Support

Participants ~~were asked to what extent they felt supported by the following individuals, organizations, or authorities~~assessed perceived support from: family, friends and social network, neighbors, employer, authorities, day-care centers, schools, church or religious community, primary care physician, hospitals (0=little or no support; 3=strong support). To compute the score, we summed up the ratings.

Infection rate

The cases per 100,000 inhabitants in the participants’ resident canton (state) were used [30~~16~~].

Agreement with containment measures

We asked the participants whether they thought that the measures were (a) not at all strict enough (b) not strict enough (c) just right (d) too strict (e) much too strict. For the analyses, the categories (a) and (e) were combined as “no agreement at all,” and the categories (b) and (d) were combined as “no agreement.” These combined categories were contrasted with (c) “agreement.”

Seeing positive aspects

We asked the respondents whether there were any positive aspects for them in the current situation (1=No; 2=Yes).

Participants' individual situation and demographics

We assessed ~~the following:~~ sex, age, education, type of household, having kids, employment, working in healthcare or essential shops with customer interaction, part of a risk group, media consumption, and estimated length of the crisis.

Data Availability

Replication data are available on the Open Science Framework [~~17~~31], https://osf.io/grvwa/

References

1. Brooks SK, Webster RK, Smith LE, Woodland L, Wessely S, Greenberg N, et al. The psychological impact of quarantine and how to reduce it: rapid review of the evidence. The Lancet. 2020;395(10227):912-20.
2. Pfefferbaum B, North CS. Mental Health and the Covid-19 Pandemic. N Engl J Med. 2020;383:510-512.
3. Fiorillo A, Gorwood P. The consequences of the COVID-19 pandemic on mental health and implications for clinical practice. Eur. Psychiatry. 2020;63(1):e32,1-2.
4. Liu S, Lithopoulos A, Zhang CQ, Garcia-Barrera MA, Rhodes RE. Personality and perceived stress during COVID-19 pandemic: Testing the mediating role of perceived threat and efficacy. Pers Individ Dif. 2021;168:110351. Epub 2020 Aug 21.
5. De Berardis D, Fornaro M, Orsolini L, Valchera A, Carano A, Vellante F et al. Alexithymia and suicide risk in psychiatric disorders: a mini-review. Front Psychiatry. 2017;8:148.

6. Pompili M, Innamorati M, Lamis DA, Erbuto D, Venturini P, Ricci F, Serafini G, Amore M, Girardi P. The associations among childhood maltreatment, "male depression" and suicide risk in psychiatric patients. Psychiatry Res. 2014;220(1-2):571-8.

~~4.~~7. Limcaoco RSG, Mateos EM, Fernandez JM, Roncero C. Anxiety, worry and perceived stress in the world due to the COVID-19 pandemic, March 2020. Preliminary results. medRxiv [Preprint]. 2020 [cited 2020 Jun 2]. Available from: https://www.medrxiv.org/content/10.1101/2020.04.03.20043992v1

~~5.~~8. Pellecchia U, Crestani R, Decroo T, Van den Bergh R, Al-Kourdi Y. Social Consequences of Ebola Containment Measures in Liberia. PLoS One. 2015;10(12):e0143036.

9. Varshney M, Parel JT, Raizada N, Sarin SK. Initial psychological impact of COVID-19 and its correlates in Indian Community: An online (FEEL-COVID) survey. PLoS One. 2020;15(5):e0233874.

~~6.~~10. Santiago CD, Wadsworth ME, Stump J. Socioeconomic status, neighborhood disadvantage, and poverty-related stress: Prospective effects on psychological syndromes among diverse low-income families. J Econ Psychol. 2011;32(2), 218-230.

~~7.~~11. Bruce ML, Hoff RA. Social and physical health risk factors for first-onset major depressive disorder in a community sample. Soc. Psychiatry. Psychiatr. Epidemiol. 1994;29(4):165-71.

~~8.~~12. Mehta K, Kramer H, Durazo-Arvizu R, Cao G, Tong L, Rao M. Depression in the US population during the time periods surrounding the great recession. J. Clin. Psychiatry. 2015;76(4):e499-504.

~~9.~~13. Wang C, Pan R, Wan X, Tan Y, Xu L, Ho CS, et al. Immediate Psychological Responses and Associated Factors during the Initial Stage of the 2019 Coronavirus Disease (COVID-19) Epidemic among the General Population in China. Int J Environ Res Public Health. 2020;17(5):1729.

~~10.~~14. Kelloway EK, Mullen J, Francis L. The Stress (of an) Epidemic. Stress and Health. 2012;28(2):91–7.

~~11.~~15. Barbisch D, Koenig KL, Shih F-Y. Is There a Case for Quarantine? Perspectives from SARS to Ebola. Disaster Med. Public Health Prep. 2015;9(5):547-53.

~~12.~~16. Wong TY, Koh GC, Cheong SK, Fong YT, Sundram M, Koh K. Concerns, Perceived Impact and Preparedness in an Avian Influenza Pandemic – a Comparative Study between Healthcare Workers in Primary and Tertiary Care. Ann Acad Med Singapore. 2008;37(2):7.

~~13.~~17. Naseem Z, Khalid R. Positive Thinking in Coping with Stress and Health outcomes: Literature Review. Journal of Research & Reflections in Education (JRRE). 2010;4(1):42-61.

~~14.~~18. Klein EM, Brähler E, Dreier M, Reinecke L, Müller KW, Schmutzer G, et al. The German version of the Perceived Stress Scale – psychometric characteristics in a representative German community sample. BMC Psychiatry. 2016;16(1):159.

~~15.~~19. Cohen S., Williamson G. Perceived stress in a probability sample of the U.S. In: S. Spacapam S, Oskamp S, editors. The social psychology of health: Claremont Symposium on Applied Social Psychology. Newbury Park, CA: Sage Publications; 1988 p. 31-67.

20. Wanklyn SG, Day DM, Hart TA, Girard TA. Cumulative childhood maltreatment and depression among incarcerated youth: impulsivity and hopelessness as potential intervening variables. Child Maltreat. 2012;17(4):306-17.

21. Guo J, Fu M, Liu D, Zhang B, Wang X, van IJzendoorn MH. Is the psychological impact of exposure to COVID-19 stronger in adolescents with pre-pandemic maltreatment experiences? A survey of rural Chinese adolescents. Child Abuse Negl. 2020;20:104667.

22. Norem JK. Defensive pessimism, optimism, and pessimism. In: Chang EC, editor, Optimism & pessimism: Implications for theory, research, and practice. Washington DC: American Psychological Association; 2001. p. 77–100.

23. Miranda R, Mennin DS. Depression, generalized anxiety disorder, and certainty in pessimistic predictions about the future. Cognit Ther Res. 2007;31(1),71-82.

24. Betsch, C. How behavioural science data helps mitigate the COVID-19 crisis. Nat Hum Behav. 2020;4:438.

25. Andrews JL, Foulkes L, Blakemore SJ. Peer Influence in Adolescence: Public-Health Implications for COVID-19. Trends Cogn Sci. 2020;24(8):585-587.

26. Tang W, Hu T, Yang L, Xu J. The role of alexithymia in the mental health problems of home-quarantined university students during the COVID-19 pandemic in China. Pers Individ Dif. 2020;165:110131.

~~16.~~27. Schneider EE, Schönfelder S, Domke-Wolf M, Wessa M. Measuring stress in clinical and nonclinical subjects using a German adaptation of the Perceived Stress Scale. Int J Clin Health Psychol. 2020;20(2):173-181.

28. Ben Loubir D, Serhier Z, Otmani N, Housbane S, Mouddene NA, Agoub M, Bennani Othmani M. Le stress perçu: validation de la traduction d'une échelle de mesure de stress en dialecte marocain [Perceived stress: validation of the translation of a stress measuring scale in Moroccan dialect]. Pan Afr Med J. 2015; 12;21:280.

~~17.~~29. Fossati A. Traduzione Italiana della scala per lo stress percepito [Italian translation of the Perceived Stress Scale]. 2010 [cited 2020 October 15]. Available from: http://www.pensierocritico.eu/files/Italian_PSS_10_with_info.pdf

~~18.~~30. Probst D. COVID-19 Information for Switzerland. 2020 [cited 2020 June 2]. Available from: https://www.corona-data.ch/

~~19.~~31. Wissmath B, Mast FW, Kraus F, Weibel D. Understanding the psychological impact of the COVID-19 pandemic and containment measures: an empirical model of stress [Dataset]. Available from: https://osf.io/grvwa/. Deposited May 13, 2020.

Supporting Information

S1 Appendix. Detailed description of materials and methods. This appendix contains all materials used in the survey, which were developed by the authors.

Reply to Reviewer 1's comments

Reviewer 1 writes that our manuscript is "well written, with clear explanations and use of data", with only one suggestion for improvement pertaining to the abstract. We have addressed this issue in the following reply.

Reviewer 1: "The only thing I would suggest rewriting is the abstract. The conclusion is alive and hard hitting, but somehow the abstract isn't. Maybe the first sentence could be stronger; 'Research suggests' is a very woolly start."

Reply: We agree with the reviewer that the abstract could be improved and have therefore revised it. The beginning of the abstract has been modified and the conclusions restructured to give weight on the impact of our findings. The revised abstract now reads as follows:

> "Epidemics such as COVID-19 and corresponding containment measures are assumed to cause psychological stress. In a survey during the lockdown in Switzerland (n=1565), we found substantially increased levels of stress in the population. In particular, individuals who did not agree with the containment measures, as well as those who saw nothing positive in the crisis, experienced high levels of stress. In contrast, individuals who are part of a risk group or who are working in healthcare or in essential shops experienced similar stress levels as the general public. The psychological mechanisms that determine stress, caused by the COVID-19 pandemic and containment measures, are not yet clear. Thus, we conducted a path analysis to gain a deeper understanding of the psychological mechanisms that lead to stress. Experiencing fear of the disease is a key driver for being worried. Our model further shows that worries about the individual, social, and economic consequences of the crisis, strongly boost stress. The infection rate in the canton (i.e. state) of residence also contributes to stress. Positive thinking and perceived social, organizational, and governmental support mitigate worries and stress. Our findings indicate that containment measures increase worries and stress, especially for those who feel that these measures are either not sufficient or go too far. Thus, highlighting positive aspects of the crisis and convincing people of the effectiveness and necessity of mitigation measures can, not only promote compliance, but also reduce stress. Our model suggests that people who feel protected by the authorities have fewer worries, which can, in turn limit the negative impact of the crisis on mental health."

Reply to Reviewer 2's comments

Reviewer 2 states that our study is "technically-sound, well-executed […] with clear explanation of research methods and sound statistical analysis." Furthermore, he or she notes that there are no ethical concerns, that the data are available, and that the conclusions are appropriate.

Reply: We appreciate Reviewer 2's positive evaluation of our manuscript and we are grateful for his or her feedback.

Reply to Reviewer 3's comments

Reviewer 3 concludes that our study is interesting. However, he or she raises some questions and provides suggestions for improvement. In the following section, we present Reviewer 3's recommendations to improve the paper and address each point in detail.

Reviewer 3: When throughout the Introduction section, the authors correctly reported that COVID-19 evokes stress and that reduced social interactions are risk factors for mental disorders such as major depression, they could even mention the association between stress, depression and negative outcomes. In particular, the unique sensory processing patterns of individuals with major affective disorders and their relationship with psychiatric symptomatology and clinicial outcomes have been reported. Hyposensitivity or hypersensitivity may be "trait" markers of individuals with major affective disorders and interventions should refer to the individual unique sensory profiles and their behavioral and functional impact in the context of real life. Thus, given the above information, I suggest to cite, within the main text, the following papers about this topic (PMID: 28855878, 31103905, 31031655). In addition, subjects with a history of childhood maltreatment may be even at increased risk of negative outcomes such as suicidal behavior. Specifically, the relation between childhood maltreatment, depression, and suicidal behaviors has been demonstrated. Importantly, the exposure to abuse and neglect as a child may enhance the risk to develop both symptoms of depression and higher suicidal risk. Thus, in order to briefly discuss this topic (although I understand that the link between depression, childhood maltreatment, and suicidal behavior is not the main topic of the present manuscript), my suggestion is to cite, within the main text, the following additional manuscripts (PMID: 25169890; 32824995; 30551339).

Reply: We thank the reviewer for these suggestions. We write in our paper that certain individuals are particularly vulnerable to consequences of the pandemic and the containment measures. We agree with the reviewer that existing research shows that alexithymia, childhood maltreatment, as well as neuroticism, can be such risk factors. In the revised version, we mention this:

> **"**Some individuals may be more vulnerable to psychosocial consequences; risk factors include certain personality traits (e.g. neuroticism, alexithymia), mental or physical pre-existing conditions, as well as previous traumatic experiences (e.g. childhood maltreatment), or the individual's current situation, such as working in healthcare, social isolation, or poverty [4-10]" (see manuscript without track changes, p. 3, line 39ff)

We agree with the reviewer that a comprehensive exploration of the impact of childhood maltreatment and alexithymia es somewhat beyond the scope of our study, but we find the inputs useful and have incorporated them later in the discussion. Our study shows that individuals who do not see anything positive about the pandemic show strongly increased stress levels. The question arises as to how this can be explained. Since childhood maltreatment is associated with hopelessness and lack of perspective, it is possible that the experience of maltreatment in the past could play a role. We have incorporated this into the conclusion, the corresponding passage reads as follows:

> "Although seeing something positive about the crisis may be associated with the individual situation, personality traits, and the respondent's coping behavior [17], our findings indicate that recognizing positive aspects could alleviate stress. Some individuals, however, do not see anything positive about the crisis. This raises the question as to why their assessment of the situation is so negative. For example, childhood maltreatment is associated with hopelessness [20] and psychological distress [5]. A recent study in connection with COVID-19 indicates that pre-pandemic maltreatment makes people particularly vulnerable to negative psychosocial consequences of the pandemic [21]. In addition, other factors and traits – such as pessimistic thinking [e.g. 22] or generalized anxiety [e.g. 23] – could account for the lack of perspective." (see manuscript without track changes, p. 7, line 110ff)

Furthermore, it is now mentioned in the manuscript (limitation section) that it would be promising to examine the role of alexithymia in further studies (see also reply to answer to point 4).

Additional references in the revised version (in the same order as they appear in the article):

Liu S, Lithopoulos A, Zhang CQ, Garcia-Barrera MA, Rhodes RE. Personality and perceived stress during COVID-19 pandemic: Testing the mediating role of perceived threat and efficacy. Pers Individ Dif. 2021;168:110351. Epub 2020 Aug 21.

De Berardis D, Fornaro M, Orsolini L, Valchera A, Carano A, Vellante F et al. Alexithymia and suicide risk in psychiatric disorders: a mini-review. Front Psychiatry. 2017;8:148.

Pompili M, Innamorati M, Lamis DA, Erbuto D, Venturini P, Ricci F, Serafini G, Amore M, Girardi P. The associations among childhood maltreatment, "male depression" and suicide risk in psychiatric patients. Psychiatry Res. 2014;220(1-2):571-8.

Santiago, C. D., Wadsworth, M. E., & Stump, J. (2011). Socioeconomic status, neighborhood disadvantage, and poverty-related stress: Prospective effects on psychological syndromes among diverse low-income families. *Journal of Economic Psychology, 32*(2), 218-230.

Wanklyn SG, Day DM, Hart TA, Girard TA. Cumulative childhood maltreatment and depression among incarcerated youth: impulsivity and hopelessness as potential intervening variables. Child Maltreat. 2012;17(4):306-17.

Guo J, Fu M, Liu D, Zhang B, Wang X, van IJzendoorn MH. Is the psychological impact of exposure to COVID-19 stronger in adolescents with pre-pandemic maltreatment experiences? A survey of rural Chinese adolescents. Child Abuse Negl. 2020;20:104667.

Norem JK. Defensive pessimism, optimism, and pessimism. In: Chang EC, editor, Optimism & pessimism: Implications for theory, research, and practice. Washington DC: American Psychological Association; 2001. p. 77–100.

Miranda R, Mennin DS. Depression, generalized anxiety disorder, and certainty in pessimistic predictions about the future. Cognit Ther Res. 2007;31(1),71-82.

Betsch, C. How behavioural science data helps mitigate the COVID-19 crisis. *Nat Hum Behav.* 2020;4:438.

Andrews JL, Foulkes L, Blakemore SJ. Peer Influence in Adolescence: Public-Health Implications for COVID-19. Trends Cogn Sci. 2020;24(8):585-587.

Tang W, Hu T, Yang L, Xu J. The role of alexithymia in the mental health problems of home-quarantined university students during the COVID-19 pandemic in China. Pers Individ Dif. 2020;165:110131.

Reviewer 3: The most relevant aims/objectives and study hypotheses of the present study need to be better described within the main text.

Reply: We agree with the reviewer that the aim of the study could be better explained. We now describe this in more detail in the revised version. Here is the relevant text passage:

"However, little is known about the psychological mechanisms that determine individual stress in this crisis. It is still unclear which individuals are particularly affected by stress, which factors have an increasing or decreasing influence on stress, and how these factors interact. In particular, the interplay between fear of the virus, infection rate, risk factors, individual and collective resources, perception of the containment strategy, and psychological

stress has not yet been fully understood. The aim of the present study is to address these questions by using path analysis.” (see manuscript without track changes, p. 3, line 52ff)

Reviewer 3: While the authors reported that they investigated a total of 1565 individuals who completed the online survey, they did not specify how many individuals were excluded to participate. In addition, the most relevant psychometric instruments reported in the present manuscript could be described more succinctly.

Reply: The number of participants refers to those who completed the survey and did not request the deletion of their data. 215 individuals had not completed or had cancelled the survey. The data of three participants were removed since they had requested the deletion of their data after completion of the survey. This is now described in the manuscript. We reassessed the description of the psychometric instruments and made it more succinct wherever possible. However, we would like to mention that the editor (see below) has asked us to provide sufficient information regarding the survey and questionnaire items.

Reviewer 3: The major shortcomings/limitations of this paper need to be better discussed as the description of the main caveats has been not included within the main text.

Reply: We thank the reviewer for bringing this point to our attention. We have now added a passage at the end of the Conclusion section, which addresses limitations and possible future studies. We also mention that it would be useful to examine the role of alexithymia and neuroticism in more detail. The additional passage reads as follows:

> “There are some limitations to the study. First, the cross-sectional design of the study limited the ability to make inferences about the directions of causality. Second, the sample is not representative in terms of gender; as in most studies using the PSS, women are somewhat overrepresented. Third, we could not assess all individual characteristics that could have an influence on stress levels. For example, neuroticism or alexithymia could be relevant traits that make individuals particularly vulnerable to crises such as a pandemic [26]. It would be interesting to investigate the role of these characteristics in future studies.” (see manuscript without track changes, p. 9, line 145ff)

Reviewer 3: What is the take-home message of the present manuscript? While the authors reported that providing support is an important way to mitigate worries, enable coping, and reduce stress, they could provide some additional conclusive remarks about this topic. Specifically, how worries and stress might be really mitigated? How protective coping strategies can be implemented? Here, more details/information are needed.

Reply: The take-home message is that the pandemic significantly increases stress levels and that this increase depends on a number of factors, with support, communicating positive aspects, and advocacy of the authorities' measures as being particularly important. We believe that we have already clearly stated this in the manuscript. However, we agree with the reviewer that we could conclude the discussion with additional suggestions on what could be done to mitigate stress. We have included a corresponding passage in the revised manuscript:

“Taken together, to mitigate stress, authorities should explain containment measures well, highlight positive aspects of the crisis, address worries, and facilitate support. Since agreement with the containment measures may decrease stress, it is crucial that the measures are well-explained and their importance emphasized, and that measures are backed up by scientific evidence. Since the protection of others is an important motivating factor to restrict

one's own everyday life [24], the authorities could thus emphasize that compliance with containment measures helps to protect those in need of protection. Furthermore, our analyses show that support through communities, relatives, and employers seems to be key in preventing stress; thus, these support networks should be encouraged to provide help. We identified young individuals as a group experiencing high stress levels. A recent study suggests that social media are particularly well-suited to reach out to adolescents [25]. Since we show that worries are a key driver for stress, our study also provides arguments for economic support, such as stimulus checks or short-time work. In addition, our study has identified groups that are particularly affected by the crisis. This information could help to distribute resources and target efforts." (see manuscript without track changes, p. 8, line 131ff)

Reply to additional comments from the Editor

The editorial office further mentions two additional requirements that need to be addressed.

Editor: Please ensure that your manuscript meets PLOS ONE's style requirements, including those for file naming.

Reply: As requested, it is ensured that all formal requirements are met.

Editor: Please include additional information regarding the survey or questionnaire used in the study and ensure that you have provided sufficient details that others could replicate the analyses. For instance, if you developed a questionnaire as part of this study and it is not under a copyright more restrictive than CC-BY, please include a copy, in both the original language and English, as Supporting Information.

Reply: As requested, we have added some additional information in order to provide sufficient details (see appendix S1).
