# Supplementary material for: Understanding the psychological impact of the COVID-19 pandemic and containment measures: An empirical model of stress
Source: PLoS One. 2021 Jul 29;16(7):e0254883. doi: 10.1371/journal.pone.0254883 (PMC8320973; doi:10.1371/journal.pone.0254883)
Supplement: Attachment — Submitted filename: Response to reviewers.docx [file pone.0254883.s002.docx]

**Reply to Reviewer 1’s comments**

Reviewer 1 writes that our manuscript is “well written, with clear explanations and use of data”, with only one suggestion for improvement pertaining to the abstract. We have addressed this issue in the following reply.

**Reviewer 1:** “The only thing I would suggest rewriting is the abstract. The conclusion is alive and hard hitting, but somehow the abstract isn't. Maybe the first sentence could be stronger; ‘Research suggests’ is a very woolly start.”

**Reply:** We agree with the reviewer that the abstract could be improved and have therefore revised it. The beginning of the abstract has been modified and the conclusions restructured to give weight on the impact of our findings. The revised abstract now reads as follows:

“Epidemics such as COVID-19 and corresponding containment measures are assumed to cause psychological stress. In a survey during the lockdown in Switzerland (n=1565), we found substantially increased levels of stress in the population. In particular, individuals who did not agree with the containment measures, as well as those who saw nothing positive in the crisis, experienced high levels of stress. In contrast, individuals who are part of a risk group or who are working in healthcare or in essential shops experienced similar stress levels as the general public. The psychological mechanisms that determine stress, caused by the COVID-19 pandemic and containment measures, are not yet clear. Thus, we conducted a path analysis to gain a deeper understanding of the psychological mechanisms that lead to stress. Experiencing fear of the disease is a key driver for being worried. Our model further shows that worries about the individual, social, and economic consequences of the crisis, strongly boost stress. The infection rate in the canton (i.e. state) of residence also contributes to stress. Positive thinking and perceived social, organizational, and governmental support mitigate worries and stress. Our findings indicate that containment measures increase worries and stress, especially for those who feel that these measures are either not sufficient or go too far. Thus, highlighting positive aspects of the crisis and convincing people of the effectiveness and necessity of mitigation measures can, not only promote compliance, but also reduce stress. Our model suggests that people who feel protected by the authorities have fewer worries, which can, in turn limit the negative impact of the crisis on mental health.”

**Reply to Reviewer 2’s comments**

Reviewer 2 states that our study is “technically-sound, well-executed […] with clear explanation of research methods and sound statistical analysis.” Furthermore, he or she notes that there are no ethical concerns, that the data are available, and that the conclusions are appropriate.

**Reply:** We appreciate Reviewer 2’s positive evaluation of our manuscript and we are grateful for his or her feedback.

**Reply to Reviewer 3’s comments**

Reviewer 3 concludes that our study is interesting. However, he or she raises some questions and provides suggestions for improvement. In the following section, we present Reviewer 3's recommendations to improve the paper and address each point in detail.

**Reviewer 3:** When throughout the Introduction section, the authors correctly reported that COVID-19 evokes stress and that reduced social interactions are risk factors for mental disorders such as major depression, they could even mention the association between stress, depression and negative outcomes. In particular, the unique sensory processing patterns of individuals with major affective disorders and their relationship with psychiatric symptomatology and clinicial outcomes have been reported. Hyposensitivity or hypersensitivity may be "trait" markers of individuals with major affective disorders and interventions should refer to the individual unique sensory profiles and their behavioral and functional impact in the context of real life. Thus, given the above information, I suggest to cite, within the main text, the following papers about this topic (PMID: 28855878, 31103905, 31031655). In addition, subjects with a history of childhood maltreatment may be even at increased risk of negative outcomes such as suicidal behavior. Specifically, the relation between childhood maltreatment, depression, and suicidal behaviors has been demonstrated. Importantly, the exposure to abuse and neglect as a child may enhance the risk to develop both symptoms of depression and higher suicidal risk. Thus, in order to briefly discuss this topic (although I understand that the link between depression, childhood maltreatment, and suicidal behavior is not the main topic of the present manuscript), my suggestion is to cite, within the main text, the following additional manuscripts (PMID: 25169890; 32824995; 30551339).

**Reply:** We thank the reviewer for these suggestions. We write in our paper that certain individuals are particularly vulnerable to consequences of the pandemic and the containment measures. We agree with the reviewer that existing research shows that alexithymia, childhood maltreatment, as well as neuroticism, can be such risk factors. In the revised version, we mention this:

**“**Some individuals may be more vulnerable to psychosocial consequences; risk factors include certain personality traits (e.g. neuroticism, alexithymia), mental or physical pre-existing conditions, as well as previous traumatic experiences (e.g. childhood maltreatment), or the individual’s current situation, such as working in healthcare, social isolation, or poverty [4-10]” (see manuscript without track changes, p. 3, line 39ff)

We agree with the reviewer that a comprehensive exploration of the impact of childhood maltreatment and alexithymia es somewhat beyond the scope of our study, but we find the inputs useful and have incorporated them later in the discussion. Our study shows that individuals who do not see anything positive about the pandemic show strongly increased stress levels. The question arises as to how this can be explained. Since childhood maltreatment is associated with hopelessness and lack of perspective, it is possible that the experience of maltreatment in the past could play a role. We have incorporated this into the conclusion, the corresponding passage reads as follows:

“Although seeing something positive about the crisis may be associated with the individual situation, personality traits, and the respondent’s coping behavior [17], our findings indicate that recognizing positive aspects could alleviate stress. Some individuals, however, do not see anything positive about the crisis. This raises the question as to why their assessment of the situation is so negative. For example, childhood maltreatment is associated with hopelessness [20] and psychological distress [5]. A recent study in connection with COVID-19 indicates that pre-pandemic maltreatment makes people particularly vulnerable to negative psychosocial consequences of the pandemic [21]. In addition, other factors and traits – such as pessimistic thinking [e.g. 22] or generalized anxiety [e.g. 23] – could account for the lack of perspective.” (see manuscript without track changes, p. 7, line 110ff)

Furthermore, it is now mentioned in the manuscript (limitation section) that it would be promising to examine the role of alexithymia in further studies (see also reply to answer to point 4).

Additional references in the revised version (in the same order as they appear in the article):

Liu S, Lithopoulos A, Zhang CQ, Garcia-Barrera MA, Rhodes RE. Personality and perceived stress during COVID-19 pandemic: Testing the mediating role of perceived threat and efficacy. Pers Individ Dif. 2021;168:110351. Epub 2020 Aug 21.

De Berardis D, Fornaro M, Orsolini L, Valchera A, Carano A, Vellante F et al. Alexithymia and suicide risk in psychiatric disorders: a mini-review. Front Psychiatry. 2017;8:148.

Pompili M, Innamorati M, Lamis DA, Erbuto D, Venturini P, Ricci F, Serafini G, Amore M, Girardi P. The associations among childhood maltreatment, "male depression" and suicide risk in psychiatric patients. Psychiatry Res. 2014;220(1-2):571-8.

Santiago, C. D., Wadsworth, M. E., & Stump, J. (2011). Socioeconomic status, neighborhood disadvantage, and poverty-related stress: Prospective effects on psychological syndromes among diverse low-income families*. Journal of Economic Psychology, 32*(2), 218-230.

Wanklyn SG, Day DM, Hart TA, Girard TA. Cumulative childhood maltreatment and depression among incarcerated youth: impulsivity and hopelessness as potential intervening variables. Child Maltreat. 2012;17(4):306-17.

Guo J, Fu M, Liu D, Zhang B, Wang X, van IJzendoorn MH. Is the psychological impact of exposure to COVID-19 stronger in adolescents with pre-pandemic maltreatment experiences? A survey of rural Chinese adolescents. Child Abuse Negl. 2020;20:104667.

Norem JK. Defensive pessimism, optimism, and pessimism. In: Chang EC, editor, Optimism & pessimism: Implications for theory, research, and practice*.* Washington DC: American Psychological Association; 2001. p. 77–100.

Miranda R, Mennin DS. Depression, generalized anxiety disorder, and certainty in pessimistic predictions about the future. Cognit Ther Res. 2007;31(1),71-82.

Betsch, C. How behavioural science data helps mitigate the COVID-19 crisis. *Nat Hum Behav.* 2020;4:438.

Andrews JL, Foulkes L, Blakemore SJ. Peer Influence in Adolescence: Public-Health Implications for COVID-19. Trends Cogn Sci. 2020;24(8):585-587.

Tang W, Hu T, Yang L, Xu J. The role of alexithymia in the mental health problems of home-quarantined university students during the COVID-19 pandemic in China. Pers Individ Dif. 2020;165:110131.

**Reviewer 3:** The most relevant aims/objectives and study hypotheses of the present study need to be better described within the main text.

**Reply:** We agree with the reviewer that the aim of the study could be better explained. We now describe this in more detail in the revised version. Here is the relevant text passage:

**“**However, little is known about the psychological mechanisms that determine individual stress in this crisis. It is still unclear which individuals are particularly affected by stress, which factors have an increasing or decreasing influence on stress, and how these factors interact. In particular, the interplay between fear of the virus, infection rate, risk factors, individual and collective resources, perception of the containment strategy, and psychological stress has not yet been fully understood. The aim of the present study is to address these questions by using path analysis.” (see manuscript without track changes, p. 3, line 52ff)

**Reviewer 3:** While the authors reported that they investigated a total of 1565 individuals who completed the online survey, they did not specify how many individuals were excluded to participate. In addition, the most relevant psychometric instruments reported in the present manuscript could be described more succinctly.

**Reply:** The number of participants refers to those who completed the survey and did not request the deletion of their data. 215 individuals had not completed or had cancelled the survey. The data of three participants were removed since they had requested the deletion of their data after completion of the survey. This is now described in the manuscript. We reassessed the description of the psychometric instruments and made it more succinct wherever possible. However, we would like to mention that the editor (see below) has asked us to provide sufficient information regarding the survey and questionnaire items.

**Reviewer 3:** The major shortcomings/limitations of this paper need to be better discussed as the description of the main caveats has been not included within the main text.

**Reply:** We thank the reviewer for bringing this point to our attention. We have now added a passage at the end of the Conclusion section, which addresses limitations and possible future studies. We also mention that it would be useful to examine the role of alexithymia and neuroticism in more detail. The additional passage reads as follows:

“There are some limitations to the study. First, the cross-sectional design of the study limited the ability to make inferences about the directions of causality. Second, the sample is not representative in terms of gender; as in most studies using the PSS, women are somewhat overrepresented. Third, we could not assess all individual characteristics that could have an influence on stress levels. For example, neuroticism or alexithymia could be relevant traits that make individuals particularly vulnerable to crises such as a pandemic [26]. It would be interesting to investigate the role of these characteristics in future studies.” (see manuscript without track changes, p. 9, line 145ff)

**Reviewer 3:** What is the take-home message of the present manuscript? While the authors reported that providing support is an important way to mitigate worries, enable coping, and reduce stress, they could provide some additional conclusive remarks about this topic. Specifically, how worries and stress might be really mitigated? How protective coping strategies can be implemented? Here, more details/information are needed.

**Reply:** The take-home message is that the pandemic significantly increases stress levels and that this increase depends on a number of factors, with support, communicating positive aspects, and advocacy of the authorities' measures as being particularly important. We believe that we have already clearly stated this in the manuscript. However, we agree with the reviewer that we could conclude the discussion with additional suggestions on what could be done to mitigate stress. We have included a corresponding passage in the revised manuscript:

“Taken together, to mitigate stress, authorities should explain containment measures well, highlight positive aspects of the crisis, address worries, and facilitate support. Since agreement with the containment measures may decrease stress, it is crucial that the measures are well-explained and their importance emphasized, and that measures are backed up by scientific evidence. Since the protection of others is an important motivating factor to restrict one's own everyday life [24], the authorities could thus emphasize that compliance with containment measures helps to protect those in need of protection. Furthermore, our analyses show that support through communities, relatives, and employers seems to be key in preventing stress; thus, these support networks should be encouraged to provide help. We identified young individuals as a group experiencing high stress levels. A recent study suggests that social media are particularly well-suited to reach out to adolescents [25]. Since we show that worries are a key driver for stress, our study also provides arguments for economic support, such as stimulus checks or short-time work. In addition, our study has identified groups that are particularly affected by the crisis. This information could help to distribute resources and target efforts.” (see manuscript without track changes, p. 8, line 131ff)

**Reply to additional comments from the Editor**

The editorial office further mentions two additional requirements that need to be addressed.

**Editor:** Please ensure that your manuscript meets PLOS ONE's style requirements, including those for file naming.

**Reply:** As requested, it is ensured that all formal requirements are met.

**Editor:** Please include additional information regarding the survey or questionnaire used in the study and ensure that you have provided sufficient details that others could replicate the analyses. For instance, if you developed a questionnaire as part of this study and it is not under a copyright more restrictive than CC-BY, please include a copy, in both the original language and English, as Supporting Information.

**Reply:** As requested, we have added some additional information in order to provide sufficient details (see appendix S1).
