# Supplementary material for: Understanding the psychological impact of the COVID-19 pandemic and containment measures: An empirical model of stress
Source: PLoS One. 2021 Jul 29;16(7):e0254883. doi: 10.1371/journal.pone.0254883 (PMC8320973; doi:10.1371/journal.pone.0254883)
Supplement: Attachment — Submitted filename: Response to reviewers.docx [file pone.0254883.s004.docx]

**Reply to Reviewer 3’s comments**

According to Reviewer 3, all comments have been addressed. He states: “In the revised manuscript, the authors addressed most of the major questions raised by Reviewers improving both the main structure and quality of the present paper. I have no further additional comments.“

**Reply:** We appreciate Reviewer 3’s positive evaluation and we are thankful for his or her positive feedback.

**Reply to Reviewer 4’s comments**

Reviewer 4 raises some questions and concerns and provides suggestions for improvement. In the following paragraphs, we present Reviewer 4's recommendations to improve the paper and address each point in detail.

**Comment 1:**

Add a section of Statistical Analysis: include information related to statistical analysis, type of variable and data presentation, processing, program used for data analysis and version and manufacturing company.

what program was used for creation of path model, what test of significance were used, did you test for normality, and what was the selected level of significance.

I suggest adding more information regard path model, what was considered significance level for paths, it seems you choose different statistical level for the paths and the tests in table 1 (0.01 vs 0.001) elaborate more, based on what certain variable were included in the model while other were not?

**Reply:**

We thank the reviewer for bringing these aspects to our attention and agree that further information is needed. We have now added a new section, “Statistical analysis,” presenting the details of the statistical analysis and the software used to conduct the analysis. Furthermore, we clarified our choice of variables for the path model, and supplemented and corrected Table 1 with additional information about levels of significance. As suggested, we additionally added a percentages column to Table 1. The section reads as follows:

“In a first step, a one-sample z-test was conducted to compare the stress level of the study sample to a representative community sample under normal conditions [22]. Before the test was carried out, we checked whether the stress values were normally distributed: the stress scores were approximately normally distributed, as the skewness equaled 0.43 (a normal distribution can be assumed for skewness values between -0.5 and 0.5 [24]). Thus, the data allowed for z-test as well as ANOVAs (step 2 below).

In a second step, an ANOVA was carried out to compare different subgroups of the study sample. Because of multiple comparisons, Bonferroni's adjustments were made to prevent Type I error inflation (α = 0.05/14 = 0.00357). IBM SPSS Statistics for Windows, Version 25.0 was used to run this analysis [25].

In a third step, we tested how stress is related to various variables that are assumed to affect well-being in the context of the pandemic. A path analysis was conducted with stress as the target variable. The path analysis was carried out with IBM SPSS Amos, Version 25.0 [26], using the maximum likelihood method. To test for normality prior to the analysis, a descriptive approach was used. In the context of SEM or path analysis, kurtosis values of > 7 indicate a substantial deviation from normality [27, 28]. In terms of skewness values of > 3 indicate extreme levels of skewness [29]. For both skewness and kurtosis, our values were below these thresholds and thus, the use of the maximum likelihood method was appropriate. To test the significance of the path coefficients, t-tests were calculated. The following variables were included in the path model: stress, fear of COVID-19, worries, support, and infection rate. These variables were selected because previous findings suggest that they affect stress. In addition, "agreement with containment measures" as well as "positive aspects" were included, because the second step of analysis (see above) showed that these variables exert the strongest influence on stress.

We used p < 0.05 as a priori level of significance. However, we reported lower p-values when appropriate (e.g., p < 0.001). Effect sizes were reported as small, medium, or strong according to the heuristics of Eid, Gollwitzer and Schmitt [30].” (Lines 125-150)

**Comment 2:**

Follow the sequence of Introduction, Methods. Results and Discussion so the methods section has to be before the result.

**Reply:**

We agree that the requested order of sections makes it easier for the reader to understand the results section and therefore improves the readability of the manuscript. As suggested, we have changed the order of the sections, which now follows the requested order: Introduction – Materials and methods – Results – Discussion.

**Comment 3 and comment 4:**

Comment for different part of manuscript are available at attached pdf file in the comment section (less than 29 point) most of them are simple modifications, kindly correct them.

Note that the methodology section has to be clear enough so if a different investigator conducted similar study, he will get comparable result. so read it again and modify.

**Reply:**

We thank the reviewer for the thorough and detailed corrections of these aspects. We have corrected details as requested and added further information where necessary.

**Comment 5**

The question 'Following the shelter in place-orders stringently'

There is no mention for this question elsewhere in methodology and even was not discussed in the discussion section. I think it's important to have few word on this question in methodology and discussion as following shelter in order is different from just agreement with containment strategy.

**Reply:**

The description of this question has now been added to the materials and methods section (see line 114 in the revised manuscript). Although there were no significant associations between this variable and any other target variable, we agree with the reviewer that this variable differs from agreement with containment strategy and therefore deserves to be discussed. We added a new paragraph about this variable in the discussion section. This reads as follows:

“In contrast to agreement with the government’s containment strategies, there was no association between following the shelter-in-place orders stringently and perceived stress. About a third of the respondents had indicated that they did not follow the orders stringently. Previously, some COVID-19-containment measures (e.g., social distancing) were found to reduce stress, while others (e.g., more days at home) led to isolation and increased stress [35]. These findings could account for the lack of an association between these two variables in our study.” (Lines 214 - 220)

**Comment 6**

Certain points regard the approaching of the study sample has to be clarified like what platform were used and how the author make sure that the respondents will not fill up the questionnaire twice? how did they know if they were Swiss residents? as there is no mention of specific question or info indicating that thing while surveying the study group.

**Reply:**

As suggested, we have added further information about the study sample and its recruitment. Concerning the possibility of participants filling out the survey twice, we would like to point out that for privacy reasons, as little data as possible was collected about the participants. For example, no IP addresses were stored. Thus, it cannot be ruled out that people took part in the survey more than once. An examination of the data for repeated or non-human responses did not reveal any indication of such responses though. The newly added section reads as follows:

“An examination of the data for repeated or non-human responses did not reveal any indication of such responses. According to their demographic information, all participants resided in a Swiss canton (as opposed to living abroad). Further information about the sample is provided in Table 1.” (Lines 74 - 77)

**Comment 7**

Proof reading has to be done as a ref. error are found in the discussion section and some sentences need to be modified

**Reply:**

MS was proofread by native speakers again. We have revised the manuscript accordingly.
